# Supplementary material for: Experiments on the influence of spot fire and topography interaction on fire rate of spread
Source: PLoS One. 2021 Jan 7;16(1):e0245132. doi: 10.1371/journal.pone.0245132 (PMC7790231; doi:10.1371/journal.pone.0245132)

# S1 Appendix

Results from experiments in raw times (seconds) rather than relative rate of spread *R’*. Note as these are raw times, variation in fuel moisture content is not accounted for.

**S1 Table A: Summary of time in seconds from experiments for head fire and off-centre fire for three spread intervals (Line 0 - Line 1, Line 0 - Line 2, Line 0 - Line 3).**

| **Fire type** | **Hill** | **Spots** | **Line 0 - Line 1** | **Line 0 - Line 2** | **Line 0 - Line 3** |
| --- | --- | --- | --- | --- | --- |
| Head fire | Absent | 0 | 43 (35 to 52) | 58.8 (44 to 72) | 90.8 (76 to 108) |
|  | Absent | 1 | 40.4 (37 to 49) | 55.8 (49 to 69) | 91.4 (76 to 109) |
|  | Absent | 2 | 38.4 (36 to 42) | 43.4 (38 to 49) | 80.6 (65 to 99) |
|  | Present | 0 | 204 (172 to 237) | 393.6 (364 to 408) | 612.6 (562 to 674) |
|  | Present | 1 | 42.6 (32 to 57) | 213.4 (179 to 242) | 582.2 (495 to 641) |
|  | Present | 2 | 33.8 (28 to 37) | 43.8 (36 to 51) | 348.4 (314 to 370) |
| Off-centre fire | Absent | 0 | 68 (45 to 96) | 94.2 (68 to 121) | - |
|  | Absent | 1 | 68 (62 to 82) | 97 (86 to 114) | - |
|  | Absent | 2 | 66 (49 to 73) | 90.4 (67 to 106) | - |
|  | Present | 0 | 178.2 (151 to 207) | 374.2 (337 to 414) | - |
|  | Present | 1 | 156.2 (112 to 214) | 374.8 (309 to 457) | - |
|  | Present | 2 | 95.4 (79 to 109) | 219.8 (180 to 284) | - |

Values are mean (min to max).

**S1 Fig A: Boxplots summarising experiment spread times in seconds.** Top row is 3 spread intervals analysed for head fire and bottom row is 2 spread intervals for off-centre fire. Times grouped by number of spot fires (x axis) and hill present (red boxes) or absent (black boxes grey fill).


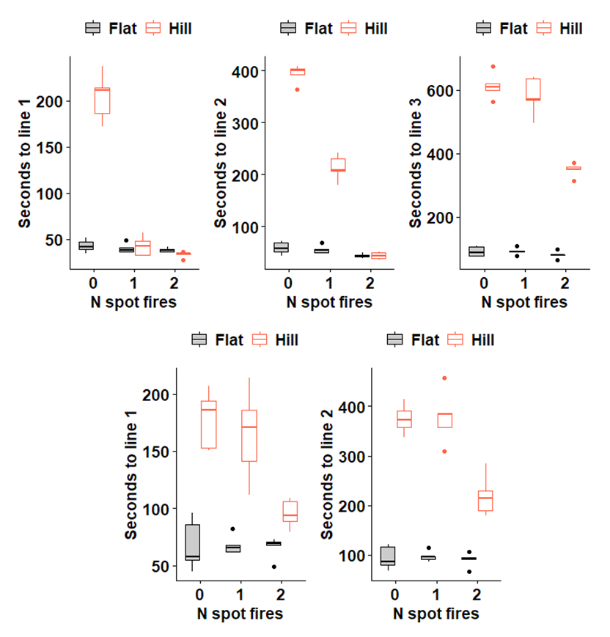

Supplement: S1 Appendix — (DOCX) [file pone.0245132.s001.docx]
