# Supplementary material for: Experiments on the influence of spot fire and topography interaction on fire rate of spread
Source: PLoS One. 2021 Jan 7;16(1):e0245132. doi: 10.1371/journal.pone.0245132 (PMC7790231; doi:10.1371/journal.pone.0245132)

# S2 Appendix

This appendix has examples of Forward Looking Infrared (FLIR) images extracted and rectified for analysis. There are 6 figures, one from each experiment configuration of spot fire number and hill presence or absence. Fig A to C are from flat fuel bed experiments (hill absent), Fig D to F are from hill present experiments. Head fire was measured between y1 and y2, and off-centre fire between y3 and y4, in the images. Note air flow measurement devices (pitot tubes) and pipes can be seen running above the fuel surface at the bottom of the flat fuel bed images (pitot data not analysed here). See S3 Video and S4 Video for example FLIR video recordings.

**S2 Fig A: Series of rectified infrared images extracted from a zero spot fire flat fuel bed experiment FLIR recording.** Images extracted at times showing (a) main fire prior to reaching any measurement line, (b) head fire has just crossed Line 1 (x1), (c) head fire has crossed Line 2 (x2), (d) head fire has crossed Line 3 (x3). Seconds after ignition are (a) 30, (b) 39, (c) 55 and (d) 86.


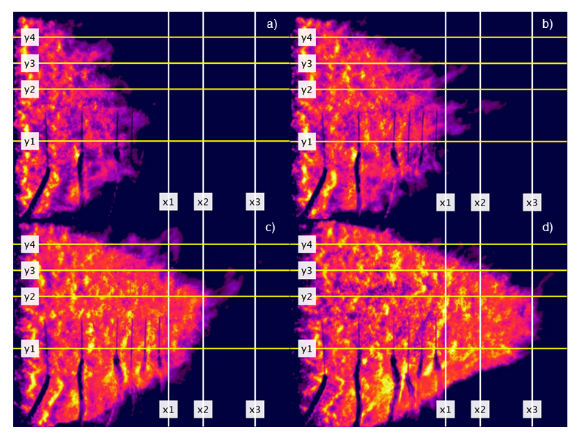


**S2 Fig B: Series of rectified infrared images extracted from a one spot fire flat fuel bed experiment FLIR recording.** Images extracted at times showing (a) main fire prior to reaching any measurement line or merging with spot fire, (b) head fire has crossed Line 1 (x1), (c) head fire has crossed Line 2 (x2), (d) head fire has crossed Line 3 (x3). Seconds after ignition are (a) 25, (b) 37, (c) 48 and (d) 90.


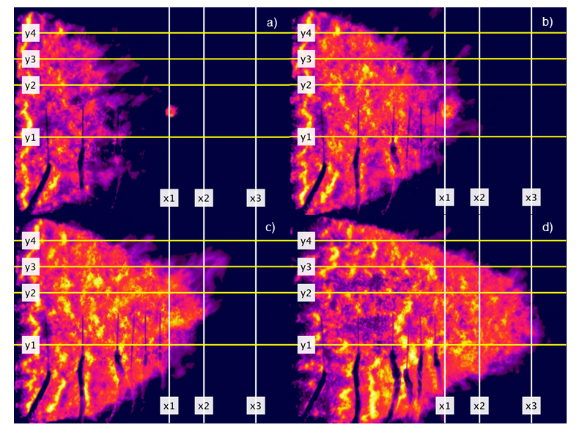


**S2 Fig C: Series of rectified infrared images extracted from a two spot fire flat fuel bed experiment FLIR recording.** Images extracted at times showing (a) main fire prior to reaching any measurement line or merging with spot fire, (b) main fire has crossed Line 1 (x1) and merged one spot fire, (c) main fire has crossed Line 2 (x2) and merged both spot fires, (d) main fire has crossed Line 3 (x3). Seconds after ignition are (a) 27, (b) 35, (c) 43 and (d) 79


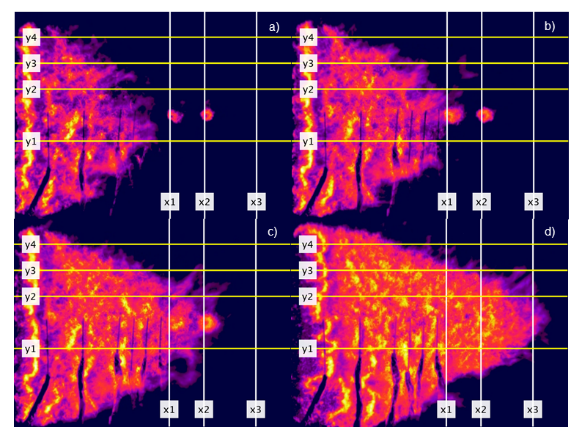


**S2 Fig D: Series of rectified infrared images extracted from a zero spot fire hill present experiment FLIR recording.** Images extracted at times showing (a) main fire prior to reaching any measurement line and approximately at ridge line, (b) head (and off-centre) fire has crossed Line 1 (x1), (c) head fire has crossed Line 2 (x2), (d) head fire has crossed Line 3 (x3). Seconds after ignition are (a) 27, (b) 244,(c) 416 and (d) 681.


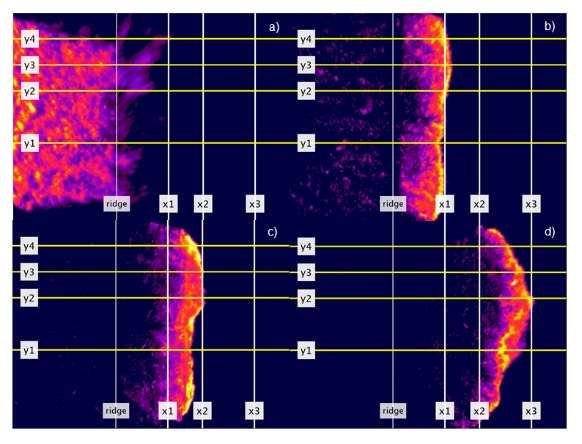


**S2 Fig E: Series of rectified infrared images extracted from a one spot fire hill present experiment FLIR recording.** Images extracted at times showing (a) main fire prior to reaching any measurement line or merging with spot fire and main fire approximately at ridge line, (b) spot fire and main fire merged, extending the new main fire (combined fire) area across Line 1 (x1) in the head fire measurement zone, (c) head fire has crossed Line 2 (x2), (d) main fire has crossed Line 3 (x3). Seconds after ignition are (a) 24, (b) 36 ,(c) 219 and (d) 584.


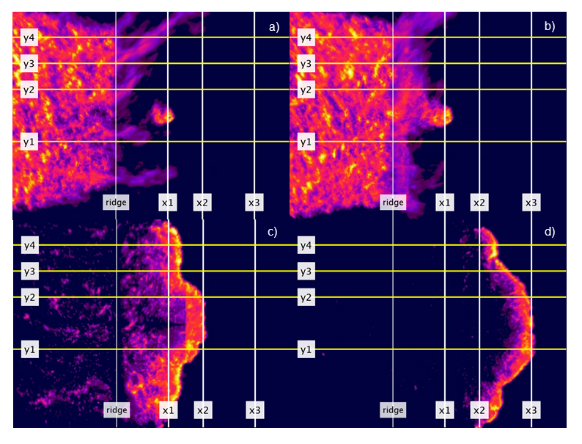


**S2 Fig F: Series of rectified infrared images extracted from a two spot fire hill present experiment FLIR recording.** Images extracted at times showing (a) main fire prior to reaching any measurement line or merging with spot fire(s) and main fire approximately at ridge line, (b) spot fire 1 and main fire merged, extending the new main fire area (combined fire) across the Line 1 (x1) in the head fire measurement zone, (c) main fire and spot fire 2 merged, extending the new main fire area (combined fire) across the Line 2 (x2) in the head fire measurement zone, (d) head fire has crossed Line 3 (x3). Seconds after ignition are (a) 22, (b) 29, (c) 43 and (d) 382.


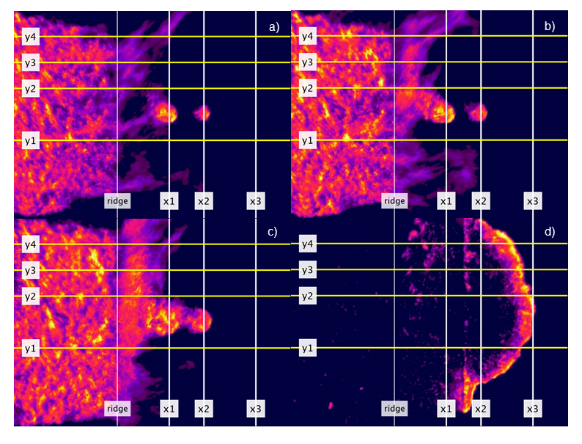

Supplement: S2 Appendix — (DOCX) [file pone.0245132.s002.docx]
