# Supplementary material for: Experiments on the influence of spot fire and topography interaction on fire rate of spread
Source: PLoS One. 2021 Jan 7;16(1):e0245132. doi: 10.1371/journal.pone.0245132 (PMC7790231; doi:10.1371/journal.pone.0245132)
Supplement: Attachment — Submitted filename: PONE-D-20-18346_reviewer.pdf [file pone.0245132.s007.pdf]

PLOS ONE

Experiments on the influence of spot fire and topography interaction on fire rate of spread

--Manuscript Draft--

**Manuscript Number:**	PONE-D-20-18346
**Article Type:**	Research Article
**Full Title:**	Experiments on the influence of spot fire and topography interaction on fire rate of spread
**Short Title:**	Experiments on the influence of spot fire and topography interaction on fire rate of spread
**Corresponding Author:**	Michael Anthony Storey<br>University of Wollongong<br>Wollongong, NSW AUSTRALIA
**Keywords:**	Spot fire; spotting; wildfire; bushfire; fire behaviour; rate of spread
**Abstract:**	Spotting is thought to increase wildfire rate of spread (ROS) and in some cases become the main mechanism for spread. The role of spotting in wildfire spread is controlled by many factors including fire intensity, number of and distance between spot fires, weather, fuel characteristics and topography. Through a set of 30 laboratory fire experiments on a 3 m x 4 m fuel bed, subject to air flow, we explored the influence of manually ignited spot fires (0, 1 or 2), the presence or absence of a model hill and their interaction on combined fire ROS (i.e. ROS incorporating main fire and merged spot fires).<br>During experiments conducted on a flat fuel bed, spot fires (whether 1 or 2) had only a small influence on combined ROS. Slowest combined ROS was recorded when a hill was present and no spot fires were ignited, because the fires crept very slowly downslope and downwind of the hill. This was up to, depending on measurement interval, 5 times slower than ROS in the flat fuel bed experiments. However, ignition of 1 or 2 spot fires (with hill present) greatly increased combined ROS to similar levels as those recorded in the flat fuel bed experiments (depending on spread interval). The effect was strongest on the head fire, where spot fires merged directly with the main fire, but significant increases in off-centre ROS were also detected.<br>Our findings suggest that under certain topographic conditions, spot fires can allow a fire to overcome the low spread potential of downslopes. Current models may underestimate wildfire ROS and fire arrival time in hilly terrain if the influence of spot fires on ROS is not incorporated into predictions.
**Order of Authors:**	Michael Anthony Storey
	Owen F Price
	Miguel Almeida
	Carlos Ribeiro
	Ross A Bradstock
	Jason J Sharples
**Additional Information:**	
**Question**	**Response**
**Financial Disclosure**<br><br>Enter a financial disclosure statement that describes the sources of funding for the work included in this submission. Review the submission guidelines for detailed requirements. View published research	This study is part of the PhD of M.S., for which he received a scholarship from the Bushfire and Natural Hazard Cooperative Research Centre (https://www.bnhcrc.com.au/) and University of Wollongong (https://www.uow.edu.au/). The funders had no role in study design, data collection and analysis, decision to publish, or preparation of the manuscript.

Powered by Editorial Manager® and ProduXion Manager® from Aries Systems Corporation

articles from *PLOS ONE* for specific examples.<br><br>This statement is required for submission and **will appear in the published article** if the submission is accepted. Please make sure it is accurate.<br><br>**Unfunded studies**<br>Enter: *The author(s) received no specific funding for this work.*<br><br>**Funded studies**<br>Enter a statement with the following details:<br>• Initials of the authors who received each award<br>• Grant numbers awarded to each author<br>• The full name of each funder<br>• URL of each funder website<br>• Did the sponsors or funders play any role in the study design, data collection and analysis, decision to publish, or preparation of the manuscript?<br>• **NO** - Include this sentence at the end of your statement: *The funders had no role in study design, data collection and analysis, decision to publish, or preparation of the manuscript.*<br>• **YES** - Specify the role(s) played.<br><br>* typeset	
**Competing Interests**<br><br>Use the instructions below to enter a competing interest statement for this submission. On behalf of all authors, disclose any competing interests that could be perceived to bias this work—acknowledging all financial support and any other relevant financial or non-financial competing interests.<br><br>This statement **will appear in the published article** if the submission is accepted. Please make sure it is accurate. View published research articles from *PLOS ONE* for specific examples.	The authors have declared that no competing interests exist.

**NO authors have competing interests**<br><br>Enter: *The authors have declared that no competing interests exist.*<br><br>**Authors with competing interests**<br><br>Enter competing interest details beginning with this statement:<br><br>*I have read the journal's policy and the authors of this manuscript have the following competing interests: [insert competing interests here]*<br><br>* typeset	
**Ethics Statement**<br><br>Enter an ethics statement for this submission. This statement is required if the study involved:<br><br>• Human participants<br>• Human specimens or tissue<br>• Vertebrate animals or cephalopods<br>• Vertebrate embryos or tissues<br>• Field research<br><br>Write "N/A" if the submission does not require an ethics statement.<br><br>General guidance is provided below. Consult the submission guidelines for detailed instructions. **Make sure that all information entered here is included in the Methods section of the manuscript.**	N/A

Powered by Editorial Manager® and ProduXion Manager® from Aries Systems Corporation

**Format for specific study types**<br><br>**Human Subject Research (involving human participants and/or tissue)**<br>• Give the name of the institutional review board or ethics committee that approved the study<br>• Include the approval number and/or a statement indicating approval of this research<br>• Indicate the form of consent obtained (written/oral) or the reason that consent was not obtained (e.g. the data were analyzed anonymously)<br><br>**Animal Research (involving vertebrate animals, embryos or tissues)**<br>• Provide the name of the Institutional Animal Care and Use Committee (IACUC) or other relevant ethics board that reviewed the study protocol, and indicate whether they approved this research or granted a formal waiver of ethical approval<br>• Include an approval number if one was obtained<br>• If the study involved *non-human primates*, add *additional details* about animal welfare and steps taken to ameliorate suffering<br>• If anesthesia, euthanasia, or any kind of animal sacrifice is part of the study, include briefly which substances and/or methods were applied<br><br>**Field Research**<br><br>Include the following details if this study involves the collection of plant, animal, or other materials from a natural setting:<br>• Field permit number<br>• Name of the institution or relevant body that granted permission	
**Data Availability**<br><br>Authors are required to make all data underlying the findings described fully available, without restriction, and from the time of publication. PLOS allows rare exceptions to address legal and ethical concerns. See the PLOS Data Policy and FAQ for detailed information.	Yes - all data are fully available without restriction

A Data Availability Statement describing where the data can be found is required at submission. Your answers to this question constitute the Data Availability Statement and **will be published in the article**, if accepted.<br><br>**Important:** Stating 'data available on request from the author' is not sufficient. If your data are only available upon request, select 'No' for the first question and explain your exceptional situation in the text box.<br><br>Do the authors confirm that all data underlying the findings described in their manuscript are fully available without restriction?	
**Describe where the data may be found in full sentences. If you are copying our sample text, replace any instances of XXX with the appropriate details.**<br><br>• If the data are **held or will be held in a public repository**, include URLs, accession numbers or DOIs. If this information will only be available after acceptance, indicate this by ticking the box below. For example: *All XXX files are available from the XXX database (accession number(s) XXX, XXX.*).<br>• If the data are all contained **within the manuscript and/or Supporting Information files**, enter the following: *All relevant data are within the manuscript and its Supporting Information files.*<br>• If neither of these applies but you are able to provide **details of access elsewhere**, with or without limitations, please do so. For example:<br><br>*Data cannot be shared publicly because of [XXX]. Data are available from the XXX Institutional Data Access / Ethics Committee (contact via XXX) for researchers who meet the criteria for access to confidential data.*<br><br>*The data underlying the results presented in the study are available from (include the name of the third party*	All relevant data are within the manuscript and its Supporting Information files.

and contact information or URL).<br>• This text is appropriate if the data are owned by a third party and authors do not have permission to share the data.<br><br>* typeset	
Additional data availability information:	

Powered by Editorial Manager® and ProduXion Manager® from Aries Systems Corporation

Manuscript

Click here to download Manuscript Fire_experiments_manuscript_PLOSONE_v1.docx

Experiments on the influence of spot fire and topography interaction on fire rate of spread

Michael A. Storey[1]*, Owen F. Price[1] , Miguel Almeida[2], Carlos Ribeiro[2], Ross A. Bradstock[1] , Jason J. Sharples[3]

[1] Centre for Environmental Risk Management of Bushfires, University of Wollongong, Northfields Ave, Wollongong NSW 2522, Australia.

[2] Association for the Development of Industrial Aerodynamics, Rua Pedro Hispano, 12; 3030-289 , Coimbra, Portugal

[3] School of Physical, Environmental and Mathematical Sciences, University of New South Wales (UNSW), Canberra ACT 2600, Australia.

*Corresponding author. Email: mas828@uowmail.edu.au

Keywords: Spot fire, spotting, wildfire, fire behaviour, rate of spread

Abstract

Spotting is thought to increase wildfire rate of spread (ROS) and in some cases become the main mechanism for spread. The role of spotting in wildfire spread is controlled by many factors including fire intensity, number of and distance between spot fires, weather, fuel characteristics and topography. Through a set of 30 laboratory fire experiments on a 3 m x 4 m fuel bed, subject to air flow, we explored the influence of manually ignited spot fires (0, 1 or 2), the presence or absence of a model hill and their interaction on combined fire ROS (i.e. ROS incorporating main fire and merged spot fires).

During experiments conducted on a flat fuel bed, spot fires (whether 1 or 2) had only a small influence on combined ROS. Slowest combined ROS was recorded when a hill was present and no spot fires were ignited, because the fires crept very slowly downslope and downwind of the hill. This was up to, depending on measurement interval, 5 times slower than ROS in the flat fuel bed experiments. However, ignition of 1 or 2 spot fires (with hill present) greatly increased combined ROS to similar levels as those recorded in the flat fuel bed experiments (depending on spread interval). The effect was strongest on the head fire, where spot fires merged directly with the main fire, but significant increases in off-centre ROS were also detected.

Our findings suggest that under certain topographic conditions, spot fires can allow a fire to overcome the low spread potential of downslopes. Current models may underestimate wildfire ROS and fire arrival time in hilly terrain if the influence of spot fires on ROS is not incorporated into predictions.

Introduction

Spotting has played a major role in some of the largest and most destructive wildfires on record [1-3]. In comparison to a continuous line of fire, spotting during wildfires creates complex discontinuous patterns of fire spread through the ignition of "spot fires". Spot fires ignite after small pieces of flaming or smouldering material (mostly vegetation such as bark or small branches, but also other materials such as building material) are transported by wind (e.g. ambient wind, plume) from the main fire to ignite unburnt fuels [4, 5]. Short to moderate distance spot fires generally merge with the main fire. This process is thought to increase the rate, and in some cases become the main driver (e.g. where profuse spotting is occurring), of wildfire spread [6-8].

An increase in wildfire rate of spread (ROS) likely occurs because nearby spot fires can quickly extend the fire front, i.e. as the main fire and spot fires merge, the overall downwind extent of the fire front is determined by the farthest downwind point of the original spot fire(s), which is farther downwind than if the spot fire(s) did not exist (Fig 1). The result is a faster combined ROS of the fire, with "combined" used here to mean the ROS incorporating the main fire and any merged spot fires (Fig 1). Effects from fire-fire interaction (e.g. increases in radiant heat and pyroconvective feedbacks) [9, 10] and junction zone behaviour (e.g. increased intensity/radiant heat) [11, 12] may enhance spread directly in the area of merging, but may also have wider effects that enhance spread in nearby parts of the fire.

Fig 1: Simple representation of spread of a spotting fire (Fire A) vs a non spotting fire (Fire B). Purpose is to demonstrate how spot fire merging may increase combined rate spread (ROS), even with identical main fire ROS. Black arrow is spread direction. Dotted lines are time 1 and solid lines are time 2. At time 1, the fires are the same, but Fire A has two separate spot fires (small circles) and Fire B has none. At time 2, "combined" ROS for Fire A is greater than for Fire B because Fire A includes the two merged spot fires, thus is farther downwind. T1 and T2 indicates head fire location at time 1 and time 2 respectively. Note that the spot fires are not considered when identifying the head fire location for Fire A time 1.

Although spotting may enhance the combined ROS, some spot fires may have little impact or may possibly reduce ROS by removing fuel ahead of the main fire [13, 14]. Numerous factors potentially control how spot fires influence fire spread including main and spot fire ROS (pre-merging), fire intensity and size, number of spot fires, spotting distances [15] and spot fire build-up times [14]. All of these factors are influenced by weather, fuel and topography [7, 16, 17]. Depending on local combinations of these (and other) factors, it is likely that similar levels of spotting could significantly influence the combined ROS in some wildfires but not others.

Topographic slope is recognised as an important factor influencing ROS and is usually included as a predictor in ROS models [18, 19]. Typically, compared to level ground, faster ROS is expected for fires travelling upslope and slower ROS expected for fires travelling downslope [20-22]. However, discontinuous spread via spotting has the potential to overcome the effects of slope [7, 23]. This is because firebrands can be transported across areas of low spread potential (downslopes, wet valleys) to ignite new spot fires on the next slope or ridge, which subsequently merge with and extend the main fire (Department of Environment Land Water and Planning unpublished data)[24]. Spotting can also exacerbate instances of dynamic spread driven by interactions between wind, steep slopes and an active fire to produce rapid rates of lateral fire spread along ridges perpendicular to the predominant wind direction [25, 26].

Capturing the effect of spotting is a major limitation of most current operational fire spread models [13]. Although recent work has been done to investigate the most effective way to include spotting in ROS models [24, 27, 28], spread models typically have not considered the influence of spotting on ROS, particularly how spotting and topography (e.g. slope) may interact to influence ROS. For example, McArthur (7) refers only generally to this interaction, by suggesting that when spotting is occurring (on large fires across multiple ridge-valleys) the effect of slope is negligible [19]. A greater understanding is required if the contribution of spotting is to be more accurately reflected in ROS

model predictions. As such, there is the need for experimental studies to understand how spot fires and topography interact, and how this influences fire spread.

We conducted a series of small experimental fires in the 6 m x 8 m combustion wind tunnel at the Forest Fire Research Laboratory of ADAI (Association for the Development of Industrial Aerodynamics) in Lousã, Portugal. The experiments incorporated variation in topography and spot fire numbers – specifically, we investigated the following research questions:

- Does the presence of downwind spot fires increase the combined forward ROS of a fire?
- If so, does the effect increase with more spot fires?
- Does any effect of the spot fires depend on the presence or absence of a hill?
- Do spot fires increase the combined ROS both directly (i.e. where spot and main fires merge) and indirectly (offset from where spot and main fires merge)?

Methods

Materials and experiment configuration

A set of 30 experiments (Table 1) were conducted over six test days in the TC 3 combustion wind tunnel (Fig 2) at the Forest Fire Research Laboratory of ADAI in Lousã, Portugal. The tunnel is housed in a large semi-open building subject to ambient air conditions (temperature and humidity). The effect of two explanatory variables on combined ROS were investigated with the experiments: number of spot fires (0, 1 or 2) and presence or absence of a model hill (Figs 2, 3 and 4). All experiments were conducted under a constant air flow at 1.5 m s^{-1}.

Fig 2: Images from the TC 3 combustion wind tunnel used for the experiments. (a) Example of model hill and fuel bed set up (note image was prior to installation of rounded ends on hill). (b) View of model hill (leeward slope) during an experiment with 2 spot fires. Combustion tunnel fan outlets are in the background in both images.

Fig 3: Schematic representation of experiments with the model hill. SF1 = Spot fire 1 location, SF2 = Spot fire 2 location. Fuel bed centre y axis (not shown) runs through SF1 and SF2 (i.e. midline between y1 and y2). Grey area is pine needle fuel bed. Head fire was measured between y1 and y2, off-centre fire between y3 and y4. Both head and off-centre fire were measured at three x axis intervals (Line 0 - Line 1, Line 0 - Line 2, Line 0 - Line 3).

Fig 4: Schematic representation of the flat fuel bed experiments. SF1 = Spot fire 1 location, SF2 = Spot fire 2 location. Fuel bed centre y axis (not shown) runs through SF1 and SF2 (i.e. midline between y1 and y2). Grey area is pine needle fuel bed. Head fire was measured between y1 and y2, off-centre fire between y3 and y4. Both head and off-centre fire were measured at three x axis intervals (Line 0 - Line 1, Line 0 - Line 2, Line 0 - Line 3).

Table 1: Summary of number of experiments performed.

Hill	Spot fires	Repetitions
Hill absent: Flat fuel bed	0	5
	1	5
	2	5
Hill present: Slope 1=21º; Slope 2 (leeward)=31º	0	5
	1	5
	2	5
Total experiments		30

For all experiments main line fire was ignited and wind speed was 1.5 m s^{-1}.

Dead mature *Pinus pinaster* needles were collected and stored in bulk in the laboratory prior to the experiments. We distributed the same amount of needles for each experiment at an average depth of 4 cm across the 3 m x 4 m (12 m^2) area of the combustion wind tunnel’s working surface (6 m x 8

m; Figs 3 and 4). For each experiment we used 0.8 kg per m^2, or 9.6 kg total, dry weight of fuel (i.e. weight of fuel if all moisture was removed). As fuel was not completely dry, we adjusted the total weight (constant dry weight component + moisture weight component, which varied with relative humidity) of fuel collected for each experiment according to its fuel moisture content (measured using an A & D ML-50 moisture analyser, as in [11, 20, 29]). To ensure a consistent amount for each experiment (9.6 kg dry weight), this meant that, for example, when fuel moisture content was lower (i.e. moisture weight component a smaller proportion of total weight) a lower total fuel weight was collected. Over the 30 experiments fuel moisture content ranged between 11% and 16%, relative humidity and temperature (measured inside the laboratory) were between 30% and 79% and 19°C and 29°C, respectively.

Fifteen experiments were conducted with a flat fuel bed (hill absent) and 15 experiments had a model hill installed (hill present). For the hill present experiments, the model hill was 400 cm wide (only the centre 300 cm was covered with fuel) and had two slopes: a 150 cm uphill section of slope 21° and a 100 cm downhill section of slope 31° (Fig 3). An additional 150 cm flat section downwind of the hill was covered with fuel (i.e. fuel bed total length 400 cm, total width 300 cm; Fig 3). There were 100 cm gaps between each end of the hill and the combustion tunnel walls. Rounded ends were added to smooth the air flow around each end of the hill (Fig 2b). For the hill absent experiments, the fuel bed was simply a 300 cm width x 400 cm length flat surface (Fig 4).

A single fire line was ignited at 0 cm (Figs 3 and 4) using wool thread soaked in a mixture of petrol and diesel in each experiment. One, two or no spot fires were ignited downwind of the fire line using diesel and petrol-soaked cotton balls placed in the fuel surface. Spot fire 1 and spot fire 2 ignition locations were constant (225 cm and 275 cm downwind) and were both along the centre y axis (Figs 3 and 4). For the hill present experiments, this meant spot fire 1 was 25 cm upslope from the base of the hill (on the leeward slope) and spot fire 2 was on the flat section 25 cm downwind of the base of the hill (Fig 3). The ignition procedure was to: 1) ignite the spot fire(s) cotton balls (when present), 2)

allow the spot fire(s) to grow to approximately 10 cm diameter (a few seconds), 3) ignite the main fire line at 0 cm, 4) turn on the air flow (set to 1.5 m s^{-1} for all experiments) once all needles below the wool thread were ignited (a few seconds). Only steps 3 and 4 were required for experiments with zero spot fires. The experiment was considered ended when any part of the fire front reached the end of the fuel bed.

Fire progression was recorded using a FLIR ThermaCam SC640 infra-red camera and colour video cameras positioned on a raised platform above (off-nadir) the fires and a side-on colour video camera.

Experiments were conducted over six days with similar weather conditions in June 2019. Due to the time necessary to install the model hill and pitot tubes (i.e. air flow measurement devices, data not analysed in this paper), flat fuel bed experiments were conducted first (days 1-3), followed by hill present experiments (days 3-6). Experiments were conducted in sets of 3 (0 spot fire, 1 spot fire and 2 spot fire) with order randomized within each set.

To account for variation in fuel moisture content of the needles across the experiments, which can influence ROS, we used a relative Rate of Spread (RROS; denoted *R’*) instead of ROS (denoted *R*) for analysis. To calculate *R’*, reference tests to measure a “basic ROS” (denoted R_0 i.e. ROS of fire line spreading on a surface without wind and slope) were conducted during each test session (approx. every 3 experiments) on a 1 m x 1 m flat table covered with 0.8 kg (dry-weight equivalent) of *Pinus pinaster* needles (as in main experiments) without wind. Basic ROS (R_0) and combined ROS from the main experiments (*R*) were used to calculate relative Rate of Spread (*R’*):

$$R' = \frac{R}{R_0}$$

R' is unitless and indicates how fast a fire in experiment conditions spread relative to a fire with no wind and no slope (R_0); For example, R' = 2 means fire in the experiment spread two times faster than R_0. Note relative rate of spread R' here is identical to NDROS in Raposo (29).

Analysis

Our analysis was based on visual inspection of images that were extracted at 1 second intervals from the FLIR video recordings (see S2 Appendix for examples). We used FLIR, as opposed to the colour video, for our analysis as greater discrimination of different flame types was possible with the FLIR (e.g. above surface flame vs burning needles). As the FLIR camera was placed off-nadir, we rectified all images in MATLAB (version R2019a) prior to the visual inspection analysis, by matching a series of control points pairs in the extracted images to a standard flat plane image showing the correct fuel bed dimensions if viewed at nadir (Figs 5 and 6). From the rectified images, we measured the combined ROS (*R*) and calculated R' for three spread intervals along the length of the fuel bed (Figs 3 and 4);

- Line 0 to Line 1 (0 cm to 225 cm, in line with spot fire 1 ignition point)
- Line 0 to Line 2 (0 cm to 275 cm, in line with spot fire 2 ignition point)
- Line 0 to Line 3 (0 cm to 350 cm, 50 cm before the end of the fuel bed)

Spot fires that were still separate from the main fire line were not considered in our ROS measurements (e.g. Figs 1, 5a and 6a), but once a spot fire merged with the main fire, the merged area was considered as a single new main fire and ROS was measured incorporating the original spot fire (e.g. Figs 1, 5b, 6b and 6c).

Fig 5: Series of rectified infrared images extracted from a one spot fire flat fuel bed experiment FLIR recording. Head fire was measured between y1 and y2, off-centre fire between y3 and y4. x1=Line 1, x2 = Line 2, x3 = Line 3. Images extracted to show examples of (a) before head fire has reached any measurement line or spot fire has merged, (b) head fire has just crossed Line 1 (x1) and

spot fire 1 has merged, (c) head fire has just crossed Line 2 (x2). Seconds after ignition are (a) 25, (b) 37 and (c) 48.

Fig 6: Series of rectified infrared images extracted from a two spot fire hill-present experiment FLIR recording. Head fire was measured between y1 and y2, off-centre fire between y3 and y4. x1=Line 1, x2 = Line 2, x3 = Line 3. Images extracted to show examples of (a) head fire approximately at the ridge line and spot fires are still separate (note light purple area is flame above fuel surface), (b) the main fire and both spot fires have merged, meaning the combined fire has crossed both Line 1 and Line 2 in the head fire zone, (c) off-centre fire has crossed Line 1 (x1), (d) off-centre fire has just crossed Line 2 (x2). Seconds after ignition are (a) 27, (b) 39, (c) 117 and (d) 200. Model hill ridge line shown for reference.

The Line 0 - Line 1 and Line 0 - Line 2 intervals were chosen as we considered these best intervals to detect any effect on ROS from one and two spot fires respectively. The Line 0 - Line 3 interval was chosen to capture any downwind effects of the spot fire(s). Note there was some small variation (a few centimetres) in the placement of the ignition line (the soaked woollen thread) between experiments, but we do not expect that this substantially affected our results.

We measured both a head fire ROS, within a 75 cm wide zone between y1 and y2 in Figs 3 and 4 (the centre line of the fuel bed was halfway between y1 and y2) and an “off-centre” ROS; a 37.5 cm wide zone between y3 (75 cm from centre line) and y4 (37.5 cm in from edge; Figs 3 and 4). Off-centre ROS was measured only on the side of the fuel bed without pitot tubes and pipes (installed for a separate analysis) to avoid any confounding effects. Off-centre ROS was only measured for the Line 0 - Line 1 and Line 0 - Line 2 intervals because off-centre fire did not reach Line 3 in a sufficient number of experiments. This was because the experiment was ended once the tip of the head fire reached the end of the fuel bed, and in most instances, this was before off-centre fire had reached Line 3.

For each experiment, we visually inspected the images to record the combined ROS (i.e. incorporating main fire and merged spot fires) for each spread interval. Then, to calculate *R′*, the process was to inspect the rectified images, as follows:

1) Record the time at which the entire fire line at Line 0 (0 cm) was ignited (time zero);

2) Beginning within the 75 cm head fire zone, record the elapsed time from time zero to the fire line burning past Line 1, Line 2 and then Line 3. For these 3 spread intervals, time was recorded if pine needles beyond the line (1, 2 or 3) and within the 75 cm head fire zone were ignited. As we measured combined ROS, this meant spot fire(s) only affected recorded times once merged with the main fire (e.g. Fig 6b and c); separate un-merged spot-fires did not affect the recorded times (e.g. Fig 6a);

3) Calculate combined ROS using the recorded times and distances for the 3 spread intervals;

4) Calculate R' (i.e. divide combined ROS (R) by R_0) ;

5) The process was then repeated for the off-centre fire zone.

Times to cross each line were identified through visual interpretation of the rectified FLIR images, all performed by one person (MS). This required discriminating actual crossings (pine needles ignited across a line) from situations where long flames ahead of the fire (and above the fuel bed) were present in the images. Thus, we first cross-checked the FLIR images with the side-on colour video for a sample of 10 experiments to confirm that above fuel bed flames (lighter purple) and burning needles (reds/yellows; Fig 6) had a sufficiently different appearance in the FLIR images to allow for such discriminations using only the FLIR images.

We conducted two-way Analysis of Variance [30] to test for the effects of hill (presence or absence) and number of spot fires (0, 1 or 2) on R'. R' was log-transformed for the analysis to meet the model assumptions (normality of residuals). A total of 5 two-way ANOVAs were performed; 3 for head fire R' (i.e. R' for the Line 0 – Line 1, Line 0 – Line 2 and Line 0 - Line 3 intervals) and 2 for off-centre fire R' (R' for the Line 0 – Line 1 and Line 0 - Line 2 intervals). Post-hoc Tukey's Tests [30] and boxplots were used to identify significant differences among treatments (i.e. hill present/absent and spot fire

number combinations) and understand the direction of the effects. $p < 0.05$ was our chosen significance level but we also report $p < 0.1$.

Results

General observations

Based on general observations of fire behaviour (FLIR, in person, colour videos) during the experiments, there was a large contrast in spot fire behaviour between the hill present and absent experiments. When the hill was absent, the spot fires were still mostly small and circular prior to merging with the main fire front (Fig 5a; Figs B and C in S2 Appendix; S3 Video). However, when the hill was present, spot fires had a larger more elongated shape prior to merging, resulting from clear, predominantly upslope spread, back towards the main fire line (i.e. opposite to main air flow; Figs E and F in S2 Appendix; S4 Video).

During all the experiments, the spot fires had only a small amount of lateral spread (they remained within the 75 cm head fire measurement zone) prior to merging with the main fire. With the hill present and after merging, there was a clear downwind extension of the new combined fire, particularly for the hill-present 2 spot fire experiments (e.g. Fig 6; Fig F in S2 Appendix; S4 Video).

In all experiments, the off-centre fire crossed Line 1 after spot fire merging had occurred. However, the first part of the new combined fire to cross Line 1 (in the off-centre fire zone) was from the direct downwind progression of the original main fire line (e.g. Fig 6c). This was also the case for off-centre fire at Line 2 for zero spot fire hill-present and all flat fuel bed experiments. However, for the 2 spot fire hill-present experiments, the first part of the fire to cross Line 2 in the off-centre measurement zone had spread outwards from the original spot fire 2 burning area (e.g Fig 6d). There was occasionally a similar, but less substantial, effect in the 1 spot fire hill-present experiments.

Head fire *R′*

For the flat fuel bed experiments (hill absent), across all spread intervals measured, mean head fire *R′* ranged between 15.3 and 26.7 (i.e. 15.3 to 26.7 times faster than R_0 - ROS on flat surface without wind). In contrast, mean head fire *R′* for the hill present experiments ranged much more widely, between 2.3 and 27.3 (Table 2).

Table 2: Summary of relative rate of spread *R′* values for experiments for head fire and off-centre fire at the three spread intervals (Line 0 - Line 1, Line 0 - Line 2, Line 0 - Line 3).

Fire type	Hill	Spots	Line 0 – Line 1	Line 0 – Line 2	Line 0 – Line 3
Head fire	Absent	0	21.8 (15.3-27.9)	19.8 (13.5-27.1)	16.1 (11.5-21.4)
	Absent	1	22.2 (17-26.4)	19.7 (14.7-23.9)	15.3 (11.9-20)
	Absent	2	24.5 (19.8-28.6)	26.7 (20.8-34)	18.6 (13.1-25.3)
	Present	0	4.6 (3.7-6)	2.9 (2.4-3.5)	2.3 (2-2.6)
	Present	1	23.9 (18.6-31.1)	5.7 (4-6.6)	2.6 (2-3)
	Present	2	27.3 (22.9-31.7)	25.7 (23.6-27.9)	4.1 (3.6-5.1)
Off-centre fire	Absent	0	14.8 (8.7-21.7)	12.6 (8.4-17.5)	-
	Absent	1	13.2 (10.2-15.7)	11.3 (8.9-13.9)	-
	Absent	2	14.7 (11.4-21.6)	13.1 (9.6-19.3)	-
	Present	0	5.3 (4.1-6.8)	3 (2.5-3.7)	-
	Present	1	6.2 (4.3-8.6)	3.2 (2.7-3.8)	-
	Present	2	9.8 (7.6-11.6)	5.2 (4.4-6)	-

Values are mean (min-max). A *R′* value of 20, for example, equates to the experiments having 20 times the ROS of the basic ROS (R_0: ROS no wind and no slope). See S1 Appendix for results in raw times (seconds).

Line 0 to Line 1 head fire *R′*

Two-way ANOVAs indicated that for the Line 0 - Line 1 spread interval, head fire *R′* was affected by a significant ($p < 0.05$) interaction between hill presence and spot fire number (Fig 7, Table 3).

Fig 7: Boxplots summarising relative rate of spread *R′* from experiments. Top row is 3 spread intervals analysed for head fire and bottom row is 2 spread intervals for off-centre fire. *R′* grouped by number of spot fires (x axis) and hill present (red boxes) or absent (black boxes grey fill). See S1 Appendix for boxplots summarising raw times.

Table 3: Summary of two-way ANOVA results for effect of spot fire number, slope and interaction term on relative rate of spread *R′*.

Interval	Term	d.f.	Head fire F	Head fire *p*	Off-centre fire F	Off-centre fire *p*
**Line 0 - Line 1**	Spots	2	71.1	**< 0.001**	4.3	**0.024**
	Slope	1	40.6	**< 0.001**	53.7	**< 0.001**
	Spots x Slope	2	57.4	**< 0.001**	3.1	0.061
**Line 0 - Line 2**	Spots	2	110.4	**< 0.001**	6.4	**0.006**
	Slope	1	217.9	**< 0.001**	216.7	**< 0.001**
	Spots x Slope	2	59.1	**< 0.001**	3.2	0.058
**Line 0 - Line 3**	Spots	2	9.7	**< 0.001**		
	Slope	1	566.9	**< 0.001**		
	Spots x Slope	2	2.8	0.080		

R′ was log-transformed for analysis. Results are separate for head fire and off-centre fire and the 3 spread intervals. d.f. = degrees of freedom, F = F statistic, p = probability. Significant p (p < 0.05) in bold text.

The zero spot fire hill-present experiments (mean 4.6) had the slowest *R′* which, despite the fast initial upslope spread, was significantly slower (using Tukey's tests with significance level $p < 0.05$) than the zero spot fire flat fuel bed experiments (mean 21.8; Tables 2 and 4). However, the introduction of spot fire(s) overcame the overall slowing effect of the hill; for 1 or 2 spot fire hill-present experiments, *R′* was similar to the flat fuel bed experiments. Amongst the hill present experiments, *R′* from the 2 spot fire (mean 27.3) and 1 spot fire (mean 23.9) experiments were significantly faster than the 0 spot fire experiments (mean 4.6), although not significantly different from each other.

Table 4: Results of post-hoc Tukey's tests, separated for the 3 spread intervals and for head fire and off-centre fire.

	Head fire			Off-centre fire	
**Comparison N spots:hill or flat**	**p value Line 0 - Line 1**	**p value Line 0 - Line 2**	**p value Line 0 - Line 3**	**p value Line 0 - Line 1**	**p value Line 0 - Line 2**
1:Flat-0:Flat	1	1	1	0.999	0.995
2:Flat-0:Flat	0.878	0.136	0.839	1	0.998
0:Hill-0:Flat	**< 0.001**	**< 0.001**	**< 0.001**	**< 0.001**	**< 0.001**
1:Hill-0:Flat	0.967	**< 0.001**	**< 0.001**	**0.001**	**< 0.001**

2:Hill-0:Flat	0.378	0.192	**< 0.001**	0.302	**< 0.001**
2:Flat-1:Flat	0.963	0.185	0.690	0.994	0.931
0:Hill-1:Flat	**< 0.001**	**< 0.001**	**< 0.001**	**< 0.001**	**< 0.001**
1:Hill-1:Flat	0.996	**< 0.001**	**< 0.001**	**0.002**	**< 0.001**
2:Hill-1:Flat	0.545	0.256	**< 0.001**	0.489	**< 0.001**
0:Hill-2:Flat	**< 0.001**	**< 0.001**	**< 0.001**	**< 0.001**	**< 0.001**
1:Hill-2:Flat	1	**< 0.001**	**< 0.001**	**< 0.001**	**< 0.001**
2:Hill-2:Flat	0.946	1	**< 0.001**	0.225	**< 0.001**
1:Hill-0:Hill	**< 0.001**	**< 0.001**	0.940	0.938	0.999
2:Hill-0:Hill	**< 0.001**	**< 0.001**	**0.002**	**0.013**	**0.008**
2:Hill-1:Hill	0.833	**< 0.001**	**0.016**	0.099	**0.020**

p values are for comparisons of mean relative rate of spread *R′* between each pair of experiments configurations of number of spot fires and hill presence or absence (flat fuel bed). Statistically significant values ($p < 0.05$) in bold.

Amongst the flat fuel bed experiments, despite some small mean increases with more spot fires ignited, no significant difference in *R′* as a function of the number of spot fires was detected with the Tukey's tests (means ranged between 21.8 to 24.5).

Line 0 to Line 2 head fire *R′*

Two-way ANOVA indicated that head fire *R′* was significantly ($p < 0.05$) affected by an interaction between hill presence and spot fire number (Fig 7, Table 3).

For the flat fuel bed, there was substantial variation of *R′* within spot fire number groups (e.g. *R′* ranged 13.5 to 27.1 for zero spot fires, in raw times between 44 and 72 seconds; S1 Appendix). However, despite some evidence of an increase in mean *R′* with 2 spot fires ignited (26.7 compared to 19.7 for 1 spot fire experiments), no significant differences between spot fire levels (0, 1 or 2) were detected with the Tukey's tests (Table 4).

For hill-present experiments, *R′* depended on the number of spot fires that were ignited. Zero spot fire experiments had the slowest *R′* (mean 2.9). *R′* increased significantly with 1 spot fire (mean 5.7) and again with 2 spot fires (mean 25.7). *R′* from the 2 spot fire experiments represented an increase by a factor of 5 and a factor of 9 compared to the 1 spot fire and 0 spot fire experiments,

respectively. These results were similar to Line 0 - Line 1 results, except now 1 spot fire *R′* was slower than 2 spot fire *R′* when the hill was present.

The results indicated that the ignition of 2 spot fires could overcome the overall slowing effect of the hill. When there were zero spot fires, hill-present *R′* was significantly slower than flat fuel bed *R′*. However, when there were 2 spot fires, hill-present *R′* was not significantly different to *R′* from the 2 spot fire flat fuel bed experiments.

Line 0 to Line 3 head fire *R′*

For the Line 0 to Line 3 interval, two-way ANOVAs indicated that head fire *R′* was significantly ($p < 0.05$) affected by hill presence and spot fire number (Fig 7, Table 3). The interaction between these factors was influential ($p < 0.1$). While for the Line 0 - Line 1 and Line 0 - Line 2 intervals spot fire(s) were able to overcome the slowing effect of the hill, this disappeared for the Line 0 - Line 3 interval. Tukey's test and boxplots showed significantly slower *R′* for hill-present experiments compared to flat fuel bed experiment regardless of spot fire number (Table 4, Fig 7).

These results reflected the slow spread observed during the experiments on the flat area downwind of the model hill. However, there was still a significant increase in *R′* resulting from downstream effect of spot fires for the hill-present experiments. *R′* from the 2 spot fire hill-present experiments (mean 4.1) was significantly faster than *R′* from both the 0 and 1 spot fire (means 2.3 and 2.6). In raw times, this equates to the 2 spot fire experiments reaching Line 3 around 4 minutes earlier than the 1 spot fire experiments (S1 Appendix). There was no significant effect of spot fire number on *R′* for the flat fuel bed experiments.

Off-centre fire *R′*

Mean off-centre fire *R′* for the flat fuel bed experiments was similar between spot fire levels, ranging between 11.3 and 14.8 (Table 2). For the hill-present experiments, mean off-centre fire *R′* was slower but ranged more widely; between 3 and 9.8.

Line 0 - Line 1 off-centre fire *R′*

Off-centre fire *R′* from Line 0 - Line 1 differed significantly as a result of hill presence and spot fire number (Table 3). The interaction between these factors was influential ($p < 0.1$). Tukey's Tests (using significance level $p < 0.05$) and boxplots indicated no significant difference in *R′* depending on spot fire number for the flat fuel bed experiments (means 13.2 to 14.8; Tables 2 and 4; Fig 7). For the hill present experiments, 2 spot fire experiment *R′* (mean 9.8) was significantly faster than 0 spot fire experiment *R′* (mean 5.3), and also not significantly different to *R′* for the flat fuel bed experiments (means 13.2 to 14.8). The *R′* for 1 spot fire (mean 6.2) and zero spot fire (mean 5.3) hill present experiments were both significantly slower than all the flat fuel bed experiments.

Line 0 - Line 2 off-centre fire *R′*

Similar results were found for the Line 0 - Line 2 spread interval. Off-centre fire *R′* was significantly ($p < 0.05$) affected by hill presence and spot fire number (Table 3), though the interaction between these factors was influential ($p < 0.1$). The Tukey's Tests and boxplots indicated no significant *R′* difference depending on spot fire number for the flat fuel bed experiments (means 11.3 to 13.1; Tables 2 and 4, Fig 7). However, for the hill present experiments, *R′* from the 2 spot fire experiments (mean 5.2) was significantly faster than both 1 spot fire (mean 3.2) and 0 spot fire experiments (mean 3). Similar to the Line 0 - Line 1 results, 2 spot fires were required to create a significant increase in *R′*. Regardless of spot fire number, off-centre fire *R′* was significantly slower for the hill experiments compared to the flat fuel bed experiments.

Discussion

Our results provide strong evidence that topography can be a major factor controlling how spot fires contribute to fire spread. In our experiments, spot fires had no significant impact on relative rate of spread (*R′*) when the fuel bed was flat, but significantly increased *R′* when a hill was present. The

effects were strongest on the head fire, but a significant increase in off-centre fire *R′* was also detected (with 2 spot fires present). The largest effect was for the Line 0 - Line 2 interval when the hill was present, where 2 spot fires increased head fire *R′* by a factor of nine compared to zero spot fires.

The large contrast in the results between the flat fuel bed and hill present experiments are likely due to differences in air flow, slope, fire-fire interaction and spot fire growth prior to merging. During the flat fuel bed experiments, with no influence of a hill and a unidirectional air flow, spot fires were overrun by the main fire before they could grow large enough to significantly influence head fire or the off-centre fire *R′*. Some fire-fire interaction probably occurred, but it was not substantial enough to significantly affect *R′*.

In contrast, the model hill may have created areas of high (up windward slope) and low (down leeward slope) spread potential through combined effects of slope angle and winds. In the absence of spot fires, and despite fast initial upslope (up windward slope) spread, the very slow spread downslope (down leeward slope) and downwind of the hill resulted in very low head and off-centre fire *R′* for the 3 spread intervals. However, when spot fire(s) were introduced, they spread up the leeward slope toward the ridge and main fire to merge with the main fire, quickly extending the main fire and resulting in high combined *R′* (particularly for Line 0 - Line 1 and Line 0 - Line 2 spread intervals). What were initially separate fires (main fire and spot fire(s)) became one main fire, with the new head fire front extending to the farthest downwind point of the original spot fire (e.g. Fig F (panels b and c) in S2 Appendix). While fires are generally expected to travel faster upslope, the upslope spread of spot fires (in opposite direction to main air flow) in our experiments was likely driven by formation of a lee-slope rotor: as air flow separated from the surface once past the ridge, a circulation of air that flowed from the base to the top of the lee slope was created, similar to previous experiments in the same laboratory with similar ridge and wind conditions [29]. As spot

fires merged with and extended the main fire, it meant that the combined fire was able to overcome the low spread potential of the downslope.

We also found that having at least two spot fires present significantly increased off-centre fire R' in the hill-present experiments (no significant influence of one spot fire was detected). In comparison to head fires, there was a more indirect effect of the spot fires on off-centre fire R' measured for Line 0 - Line 1, as the spot fires did not burn directly into the off-centre fire measurement zone prior to off-centre fire crossing Line 1. Fire-fire interaction and merging of spot fires may have generated greater radiant heat, which led to more rapid drying and ignition of nearby fuels (i.e. in the off-centre measurement zone), thus faster R'. The faster off-centre fire R' measured for Line 0 - Line 2 (hill present and 2 spot fires) likely resulted from the downstream effects of higher R' for Line 0 - Line 1, but also from the outward spread of spot fire 2 (into the off-centre fire measurement zone) after it had merged with the main fire (Fig 6 and Fig F in S2 Appendix). In all the 2 spot fire hill present experiments, the fire front had a much more curved shape due the extension (and combination of downwind and outward spread) caused by the merging of the 2 spot fires. Some junction zone behaviour, where increased spread is observed between two arms of fire, may have also influenced these results [11, 12].

It is important to consider how the results from our experiments, under a narrow set of conditions, may apply to actual wildfires. A main limitation of our study is the small scale, which was necessary to isolate the conditions (wind, slope, fuel etc.) for the study. Although we have identified basic mechanisms, it is unknown exactly how our results would scale to an actual wildfire. For example, during the hill present experiments, flames shot over the ridge and extended (above the fuel bed) past Line 1 (i.e. 75 cm past ridge, above spot fire 1) and above Line 2 in some cases (i.e. 125 cm past ridge, above spot fire 2; see Fig E (panels a & b) and Fig F (panels a & b) in S2 Appendix). This would have resulted in direct heat exchange with the spot fires that may have enhanced spread and intensity. This may occur in some smaller wildfires in certain topography, but it is unlikely that

flames would shoot above spot fires once fires reach a certain size/spotting distance (e.g. a spotting distance of say 100 m would require flames of ~ 100 m). However, some supplementary experiments we conducted with only one spot fire (no fire line) found that a single spot fire still predominantly spread back upslope, suggesting that such heat exchange was not the major determinant of spot fire spread during the experiments.

The role of spotting in the spread of wildfires is likely controlled by a wider range of factors than those considered in our experiments. These include main fire ROS, spot fire build-up time, distance between spot fire and main fire [14] and fire sizes/intensities, which vary substantially in actual fires. Wildfires also commonly burn across complex topography that includes variable slope angles, multiple ridges and variable orientation of wind to local topographic features.

However, the effects and drivers described above for the hill present experiments are thought to be important in actual wildfires burning in areas with steep slopes. For example, lee-slope rotors can encompass most of the lee slope in fire prone areas [31], and modelling and observation confirm they are important to the extreme fire behaviour of vorticity-driven lateral spread [25, 26, 32]. Coupled modelling of larger scale hills suggest that spot fires on a lee slope exhibit very similar behaviour of upslope spread as occurred with the experiment spot fires [26, 32]. While lee-slope rotors are important, other wind flows may result in a different pattern of spot fire behaviour and merging, such as more laminar flows resulting in downslope winds (e.g. Foehn winds, katabatic winds) or wind flow of different orientation to the ridge [33, 34].

Our experiments demonstrated that, for some spread intervals, increasing spot fires from one to two can increase combined ROS. It could be reasonably expected that further increasing the number of spot fires (or perhaps spot fire build-up time and size) could further increase combined ROS. Wildfires can produce hundreds of individual spot fires at any one time, including mass spotting events [1, 35, 36] that could cause a fire to, as suggested by Cheney (8); “appear to be moving as a continual coalescence of spot fires”.

Unpublished data (Victorian Department of Environment, Land, Water and Planning - FLIR video and line scans) suggest that during the Wye River wildfire in 2015 (Victoria, Australia), a rapid downslope spread of ~2 km h^{-1} occurred, resulting from a fire on a ridge. This fire set off the ignition of hundreds of spot fires (including spot to spot ignitions) downslope that subsequently merged together, thus creating a new main fire that extended from the original ridge fire to the base of the hill around 2.5 km away. Although infrared video from that fire suggests lee slope rotors may not have formed, the high numbers of spot fires that merged resulted in a similar effect to that of our hill present experiments. Moreover, coupled modelling by Toivanen, Engel (37) found that the spread of the Kilmore East wildfire in 2009 (Victoria, Australia) could be reproduced only if the ignition and merging of 18 downwind spot fires was incorporated. These suggest that, considering the combined ROS, spotting and merging might be important not just over a single slope (as in our experiments), but over a much larger scale (spotting occurred up to 30 km away in the Kilmore East wildfire [1]).

Further research is needed to explore these issues and to further investigate the role of spot fires in wildfire spread. A mixture of experiments, fire spread simulation and empirical wildfire analysis could be used to research, for example, the effect of more complex terrain (e.g. multiple hills, different slope steepness and orientation to wind), different spot fire numbers/locations and different wind speeds.

Our results have important implications for wildfire spread modelling. In particular, our results suggest spotting can lead to a substantial increase in ROS despite the presence of a downward slope, resulting from a pattern of spot fire and main fire merging. Such a pattern of fire spread has been referenced in general in the literature [7] but has typically not been included in models of ROS. Some models are attempting to incorporate the effects of spotting on ROS; e.g. through stochastic processes [24, 27, 28]. However, our results suggest that the interaction between spotting and topography, where the effect of spotting on ROS varies depending on local topography, is highly

influential on fire spread. Our results also suggest that a wildfire's ROS in hilly areas cannot be modelled accurately unless spotting is accounted for implicitly (e.g. incorporating spotting wildfires in the development of empirical spread models) or explicitly (e.g. physical modelling of main and spot fires and patterns of merging).

Conclusion

Our experiments demonstrate that topography can play a substantial role in determining how spot fires contribute to the combined ROS of a fire: the combined presence of a hill and spot fires can potentially elevate ROS. Spot fires may allow head fires to overcome areas of low spread potential (downslope, sheltered area downwind of hill) through merging, along with wider effects (e.g. possible increased radiant heat) that increase off-centre fire ROS. Predictions of fire spread in a hilly landscape using models that do not incorporate the interaction between spot fire and topography may underestimate combined ROS and fire arrival time. Larger scale experiments (with wider range of conditions), empirical analysis of wildfires and coupled modelling of spread could be used to further understand the contribution of spotting to combined ROS.

Acknowledgements

Thanks to Jorge Raposo, Nuno Luís and Gonçalo Rosa that participated in the experiments.

We would like to thank the FCT-Foundation for Science and Technology for the PhD of Carlos Ribeiro with the reference (SFRH/BD/140923/2018). The provision of a PhD scholarship to Michael Storey from the Bushfire and Natural Hazards Cooperative Research Centre and University of Wollongong is gratefully acknowledged.

References

1. Cruz MG, Sullivan AL, Gould JS, Sims NC, Bannister AJ, Hollis JJ, et al. Anatomy of a catastrophic wildfire: the Black Saturday Kilmore East fire in Victoria, Australia. Forest Ecology and Management. 2012;284:269-85. doi: https://doi.org/10.1016/j.foreco.2012.02.035.
2. Rawson RP, Billing PR, Duncan SF. The 1982–83 forest fires in Victoria. Australian Forestry. 1983;46(3):163-72. doi: 10.1080/00049158.1983.10674395.
3. McLeod R. The Inquiry into the Operational Response to the January 2003 Bushfires. Australia Capital Territory Government. Canberra: 2003.
4. Albini FA, Alexander ME, Cruz MG. A mathematical model for predicting the maximum potential spotting distance from a crown fire. International Journal of Wildland Fire. 2012;21(5):609-27. doi: http://dx.doi.org/10.1071/WF11020.
5. Koo E, Pagni PJ, Weise DR, Woycheese JP. Firebrands and spotting ignition in large-scale fires. International Journal of Wildland Fire. 2010;19(7):818-43. doi: http://dx.doi.org/10.1071/WF07119.
6. Catchpole W. Fire properties and burn patterns in heterogeneous landscapes. In: Bradstock RA, Williams JE, Gill MA, editors. Flammable Australia: the fire regimes and biodiversity of a continent: Cambridge University Press; 2002. p. 49-75.
7. McArthur AG. Fire behaviour in Eucalypt forests. Department of National Development Forestry And Timber Bureau. 1967;Leaflet Number 107:36.
8. Cheney NP. Fire Behaviour. In: Gill AM, Groves RH, Noble IR, editors. Fire and the Australian Biota. 1. Canberra: Australian Academy of Science; 1981. p. 170.
9. Finney MA, McAllister SS. A review of fire interactions and mass fires. Journal of Combustion. 2011;2011. doi: https://doi.org/10.1155/2011/548328.
10. Hilton JE, Sullivan AL, Swedosh W, Sharples J, Thomas C. Incorporating convective feedback in wildfire simulations using pyrogenic potential. Environmental Modelling & Software. 2018;107:12-24. doi: https://doi.org/10.1016/j.envsoft.2018.05.009.
11. Raposo JR, Viegas DX, Xie X, Almeida M, Figueiredo AR, Porto L, et al. Analysis of the physical processes associated with junction fires at laboratory and field scales. International Journal of Wildland Fire. 2018;27(1):52-68. doi: https://doi.org/10.1071/WF16173.
12. Viegas DX, Raposo JR, Davim DA, Rossa CG. Study of the jump fire produced by the interaction of two oblique fire fronts. Part 1. Analytical model and validation with no-slope laboratory experiments. International Journal of Wildland Fire. 2012;21(7):843-56. doi: http://dx.doi.org/10.1071/WF10155.
13. Alexander ME, Cruz MG. Limitations on the accuracy of model predictions of wildland fire behaviour: A state-of-the-knowledge overview. The Forestry Chronicle. 2013;89(03):372-83.
14. Alexander ME, Cruz MG. Evaluating a model for predicting active crown fire rate of spread using wildfire observations. Canadian Journal of Forest Research. 2006;36(11):3015-28.
15. Storey MA, Price OF, Sharples JJ, Bradstock RA. Drivers of long-distance spotting during wildfires in south-eastern Australia. International Journal of Wildland Fire. 2020:-. doi: https://doi.org/10.1071/WF19124.
16. Rothermel RC. Predicting behavior and size of crown fires in the Northern Rocky Mountains. Ogden, UT: Forest Service, 1991 INT-438 Contract No.: INT-438.
17. Werth PA, Potter BE, Alexander ME, Clements CB, Cruz MG, Finney MA, et al. Synthesis of knowledge of extreme fire behavior: volume 2 for fire behavior specialists, researchers, and meteorologists. Gen Tech Rep PNW-GTR-891 Portland, OR: US Department of Agriculture, Forest Service, Pacific Northwest Research Station 258 p. 2016;891.
18. Cheney P, Sullivan A. Grassfires: fuel, weather and fire behaviour: Csiro Publishing; 2008.

19. Cruz MG, Gould JS, Alexander ME, Sullivan AL, McCaw WL, Matthews S. Empirical-based models for predicting head-fire rate of spread in Australian fuel types. Australian Forestry. 2015;78(3):118-58. doi: 10.1080/00049158.2015.1055063.

20. Viegas DX. Slope and wind effects on fire propagation. International Journal of Wildland Fire. 2004;13(2):143-56.

21. Noble IR, Gill AM, Bary GAV. McArthur's fire-danger meters expressed as equations. Australian Journal of Ecology. 1980;5(2):201-3.

22. Sullivan AL, Sharples JJ, Matthews S, Plucinski MP. A downslope fire spread correction factor based on landscape-scale fire behaviour. Environmental Modelling & Software. 2014;62:153-63. doi: https://doi.org/10.1016/j.envsoft.2014.08.024.

23. Luke RH, McArthur AG. Bush fires in Australia. Canberra: Australian Government Publishing Service; 1978. 359 p.

24. Boychuk D, Braun W, J. Kulperger R, L. Krougly Z, Stanford D. A stochastic forest fire growth model2009. 133-51 p.

25. Sharples JJ, McRae RH, Wilkes SR. Wind–terrain effects on the propagation of wildfires in rugged terrain: fire channelling. International Journal of Wildland Fire. 2012;21(3):282-96.

26. Simpson CC, Sharples JJ, Evans JP. Sensitivity of atypical lateral fire spread to wind and slope. Geophysical Research Letters. 2016;43(4):2015GL067343. doi: 10.1002/2015GL067343.

27. Kaur I, Mentrelli A, Bosseur F, Filippi J-B, Pagnini G. Turbulence and fire-spotting effects into wild-land fire simulators. Communications in Nonlinear Science and Numerical Simulation. 2016;39:300-20. doi: https://doi.org/10.1016/j.cnsns.2016.03.003.

28. Trucchia A, Egorova V, Butenko A, Kaur I, Pagnini G. RandomFront 2.3: a physical parameterisation of fire spotting for operational fire spread models – implementation in WRF-SFIRE and response analysis with LSFire+. Geosci Model Dev. 2019;12(1):69-87. doi: 10.5194/gmd-12-69-2019.

29. Raposo JR. Experimental analysis of fire spread across a two-dimensional ridge under wind conditions. International journal of wildland fire. 2015;24(7):1008-22. doi: 10.1071/WF14150.

30. R-Core-Team. R: A Language and Environment for Statistical Computing (Version 4.0.0). Vienna, Austria: R Foundation for Statistical Computing; 2020.

31. Sharples JJ, McRae RHD, Weber RO. Wind characteristics over complex terrain with implications for bushfire risk management. Environmental Modelling and Software. 2010;25(10):1099-120.

32. Simpson CC, Sharples JJ, Evans JP, McCabe MF. Large eddy simulation of atypical wildland fire spread on leeward slopes. International Journal of Wildland Fire. 2013;22(5):599-614. doi: http://dx.doi.org/10.1071/WF12072.

33. Sharples JJ. An overview of mountain meteorological effects relevant to fire behaviour and bushfire risk. International Journal of Wildland Fire. 2009;18(7):737-54.

34. Sharples JJ. Foehn Winds. In: Manzello SL, editor. Encyclopedia of Wildfires and Wildland-Urban Interface (WUI) Fires. Cham: Springer International Publishing; 2018. p. 1-7.

35. Tedim F, Leone V, Amraoui M, Bouillon C, Coughlan M, Delogu G, et al. Defining Extreme Wildfire Events: Difficulties, Challenges, and Impacts. Fire. 2018;1(1):9. PubMed PMID: doi:10.3390/fire1010009.

36. Storey MA, Price OF, Bradstock RA, Sharples JJ. Analysis of Variation in Distance, Number, and Distribution of Spotting in Southeast Australian Wildfires. Fire. 2020;3(2).

37. Toivanen J, Engel CB, Reeder MJ, Lane TP, Davies L, Webster S, et al. Coupled Atmosphere-Fire Simulations of the Black Saturday Kilmore East Wildfires With the Unified Model. Journal of Advances in Modeling Earth Systems. 2019;11(1):210-30. doi: 10.1029/2017MS001245.

Supporting Information

S1 Appendix: S1 Appendix

S2 Appendix: S2 Appendix

S3 Video: Example of a two spot fire hill absent experiment FLIR recording

S4 Video: Example of a two spot fire hill present experiment FLIR recording

S5 File: Experiment data for head fire analysis

S6 File: Experiment data for off-centre fire analysis

Click here to download Figure Fig1.tif

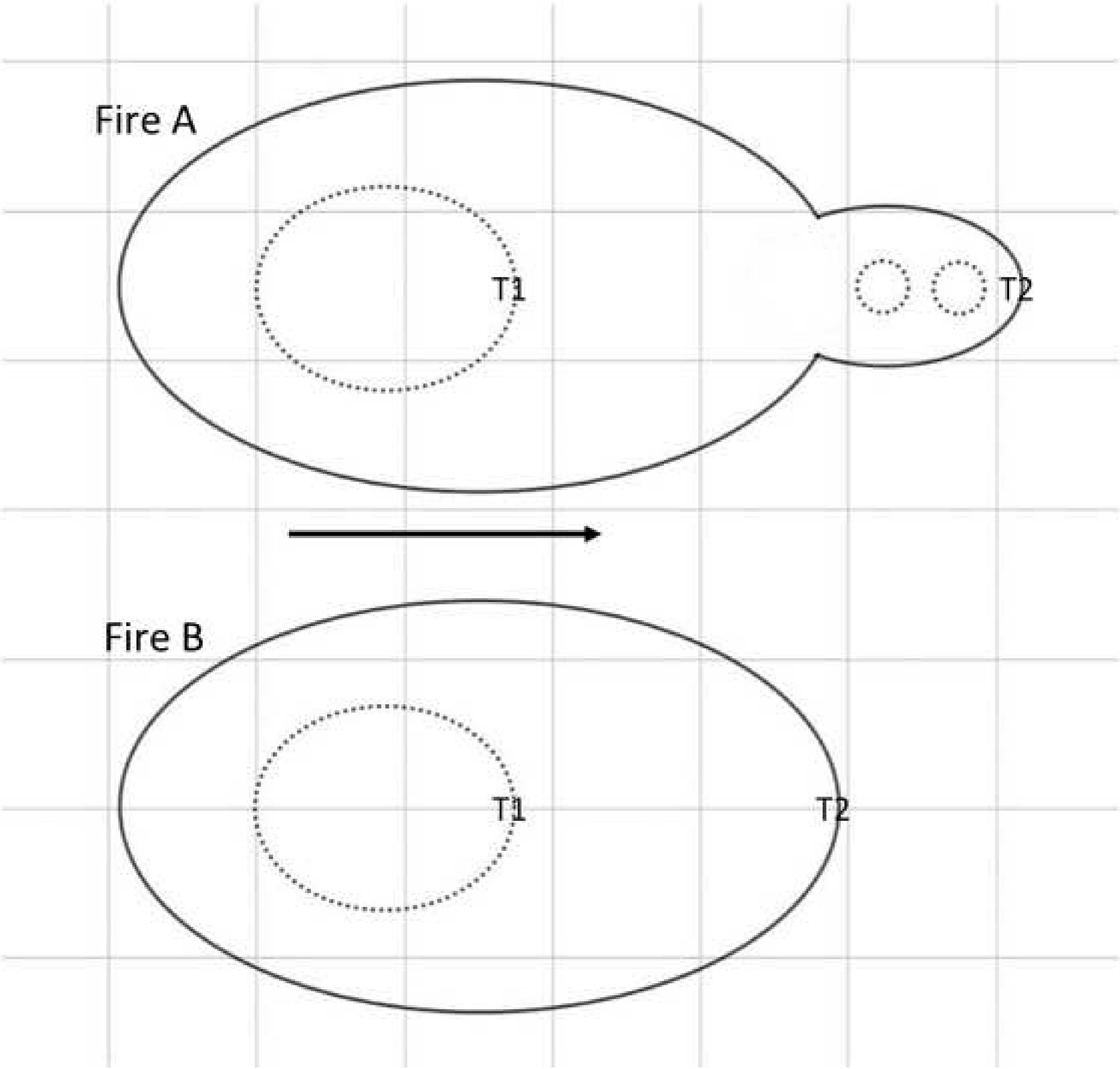

 Click here to download Figure Fig2.tif

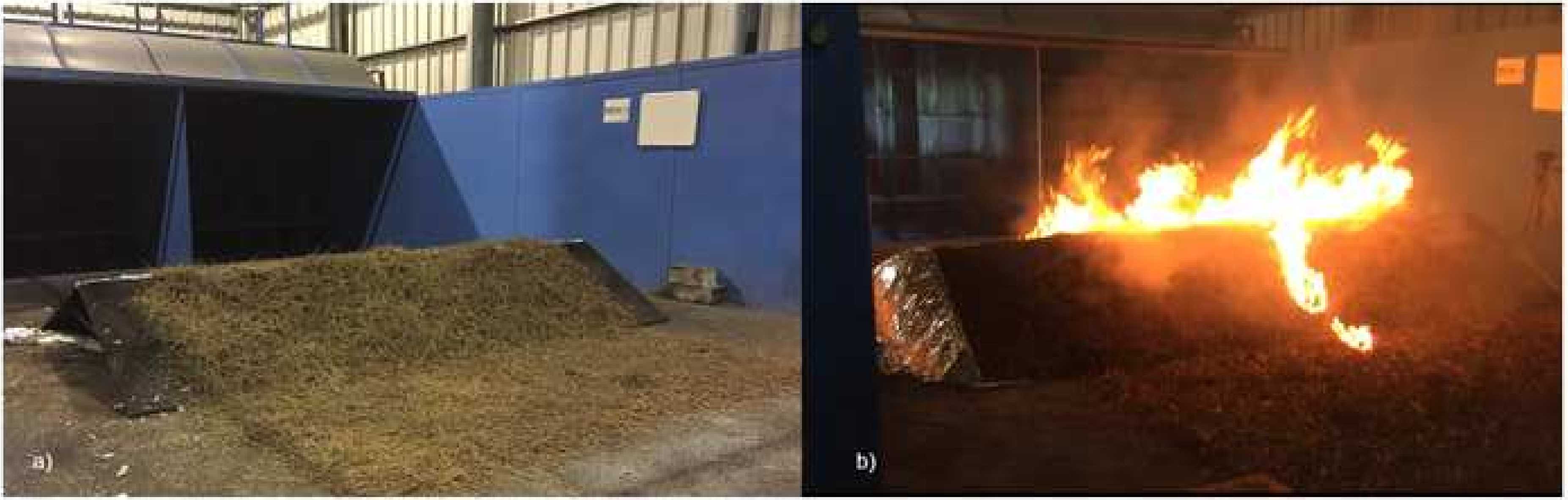

Figure

Click here to download Figure Fig3.tif

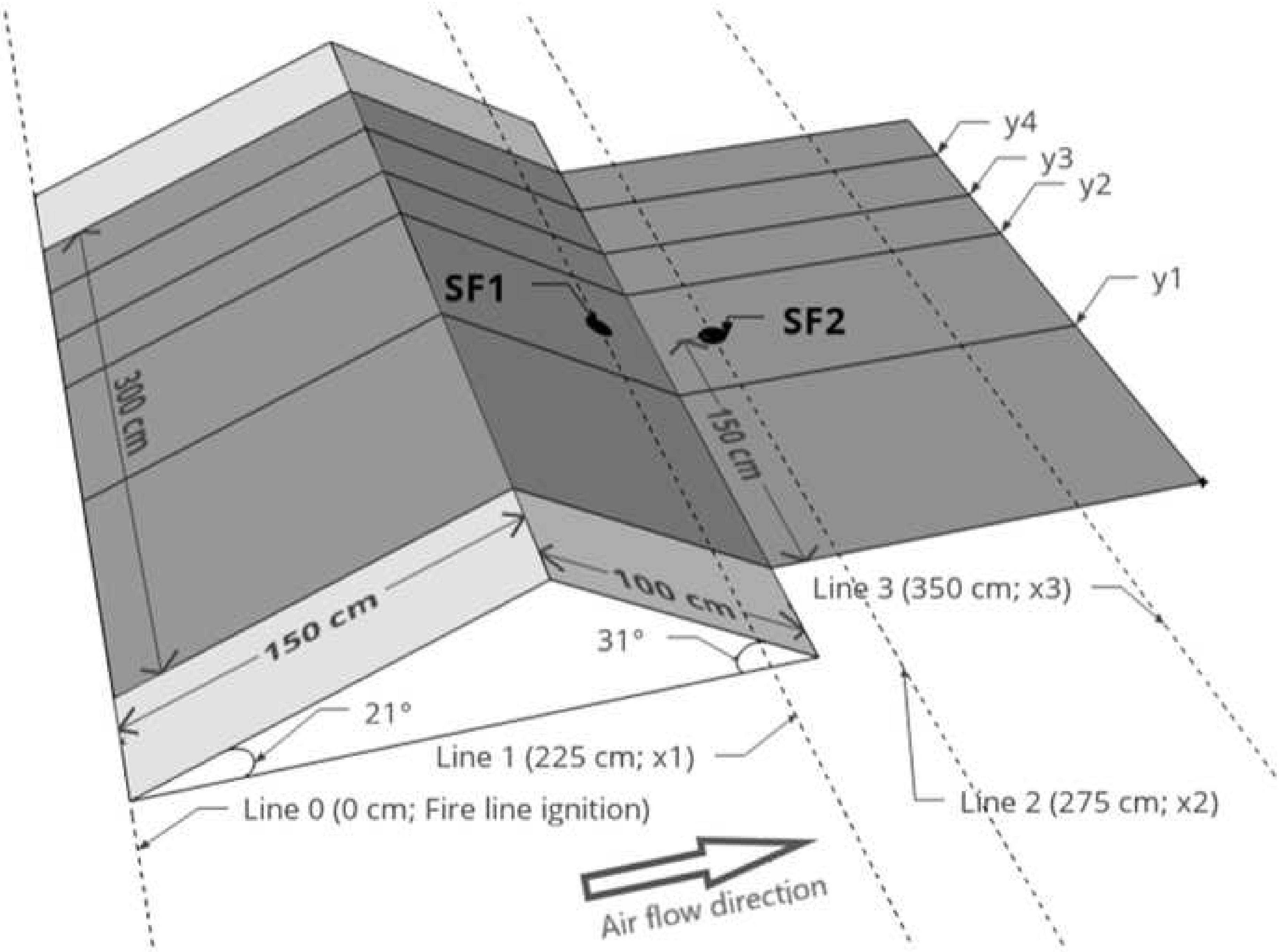

Figure

Click here to download Figure Fig4.tif

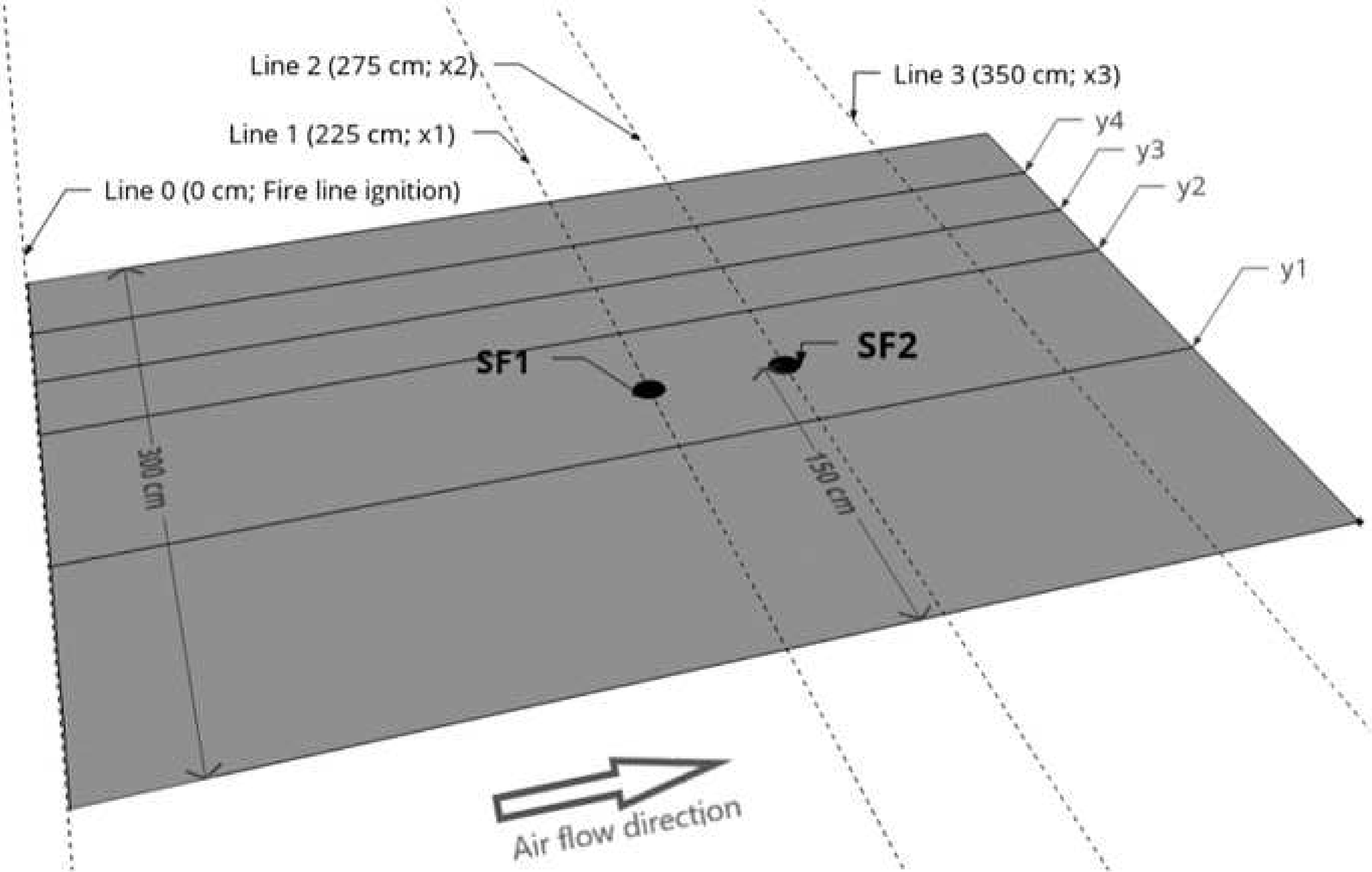

Figure

Click here to download Figure Fig5.tif

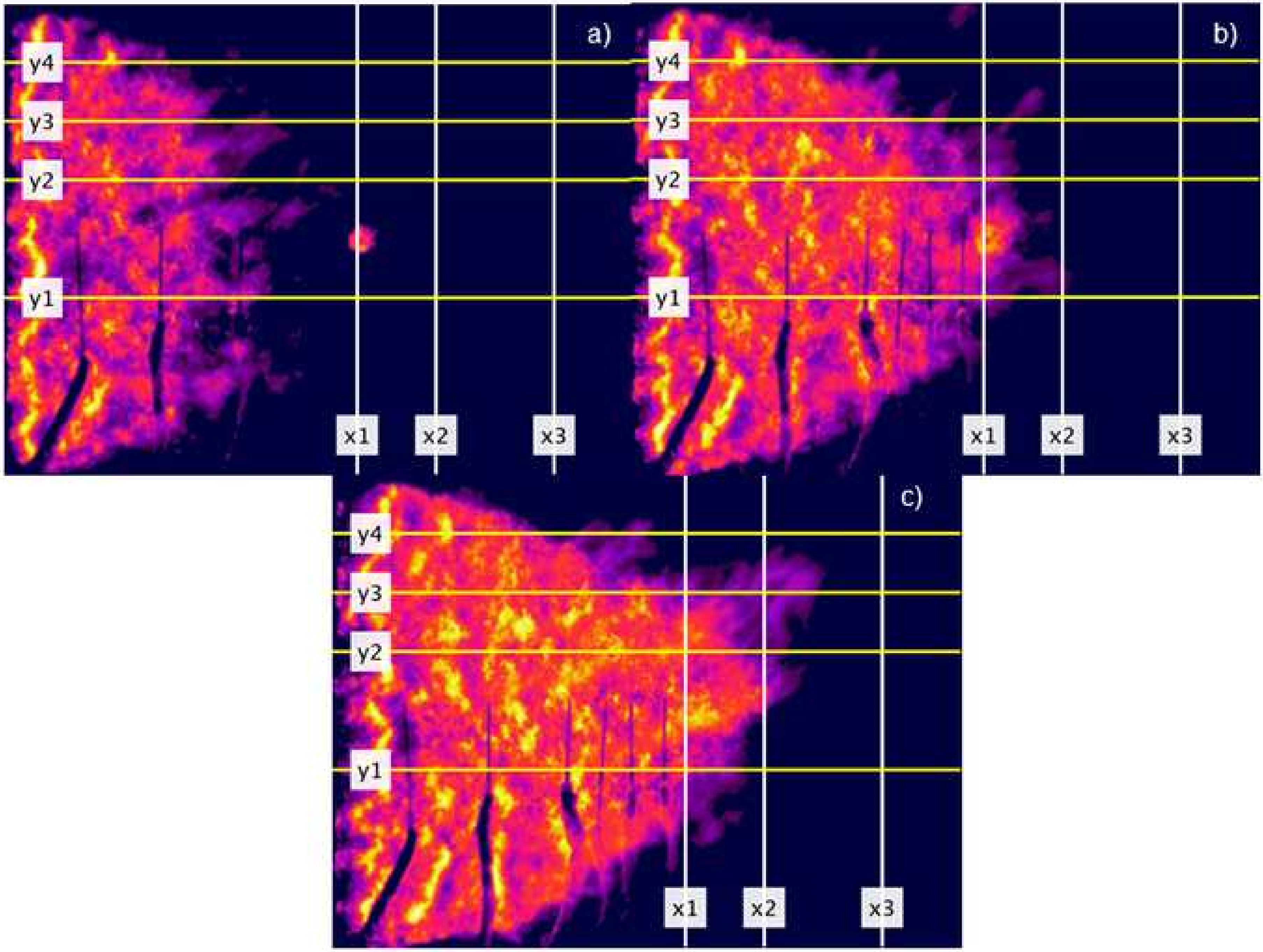

Figure

Click here to download Figure Fig6.tif

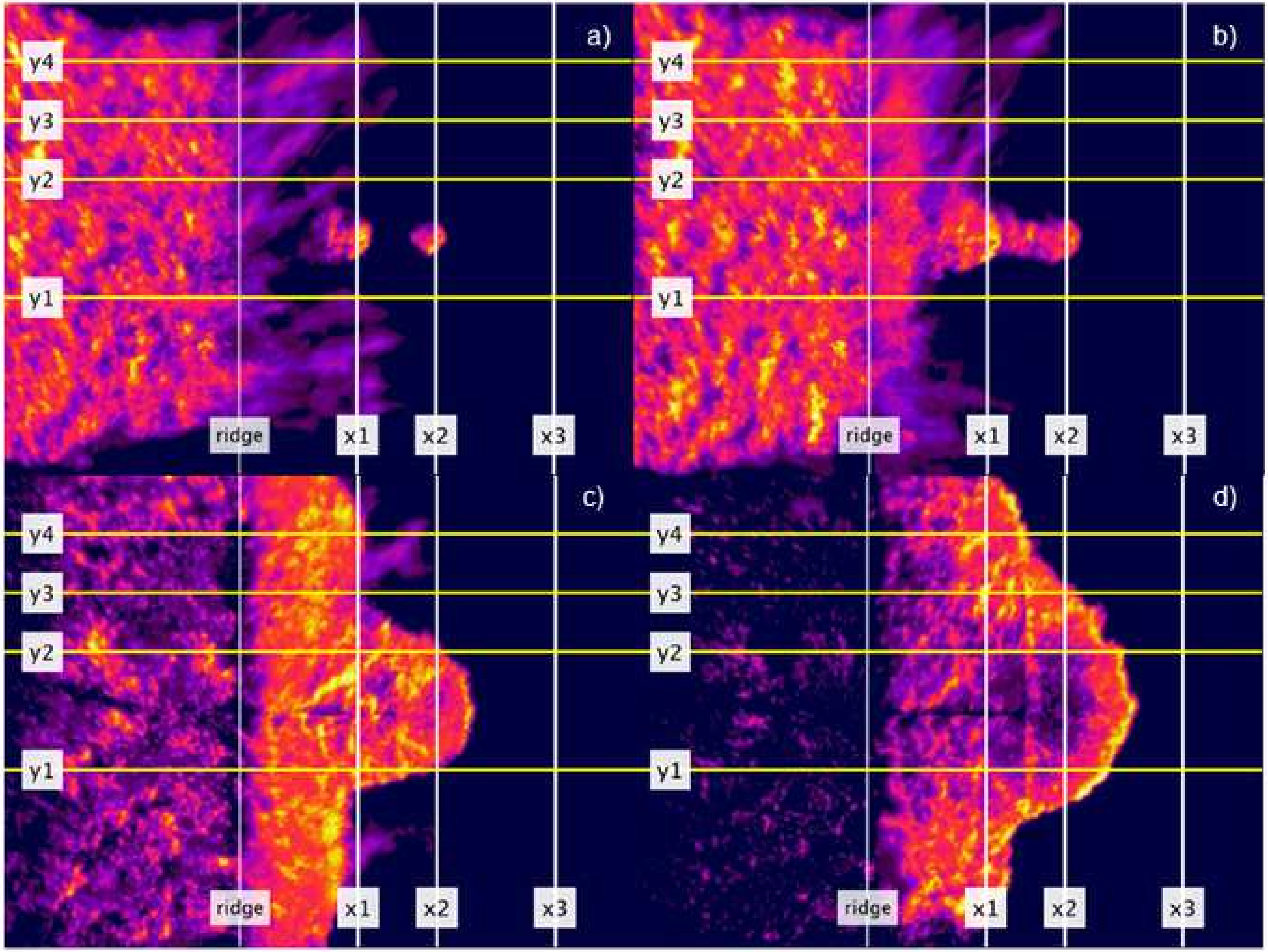

Figure

Click here to download Figure Fig7.tif

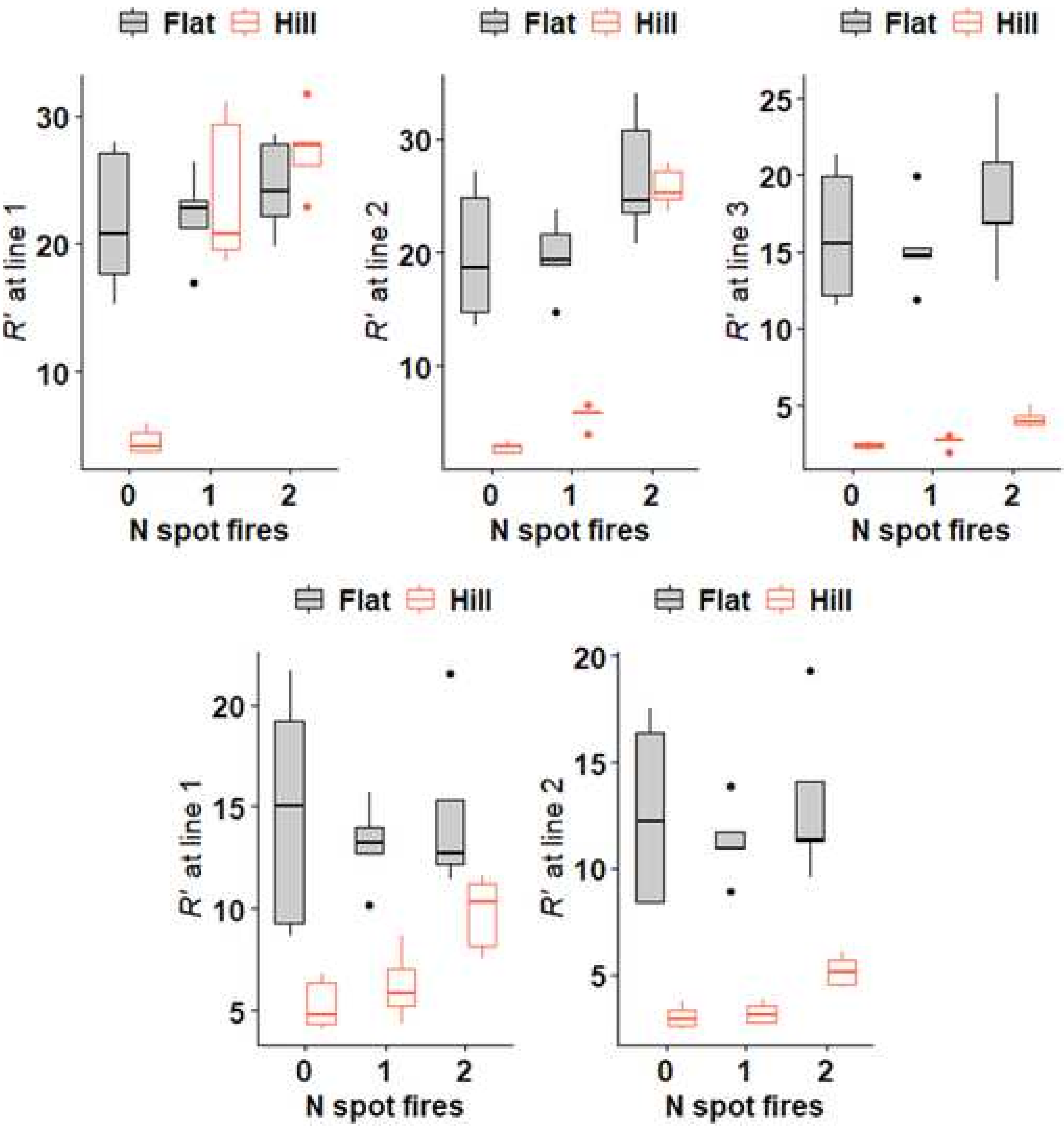

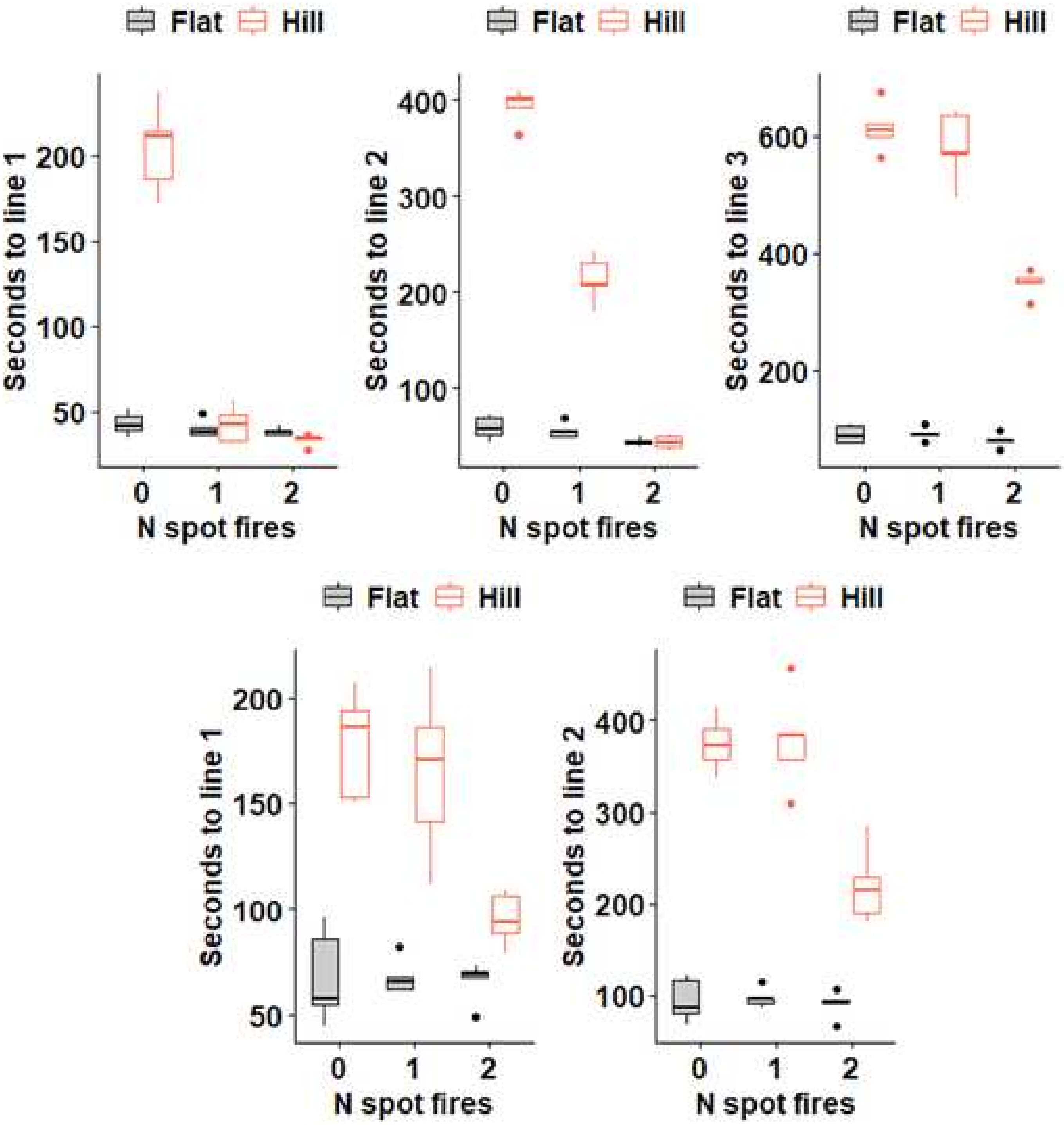
Flat
Hill
Seconds to line 1
Seconds to line 2
Seconds to line 3
N spot fires
0
1
2
50
100
150
200
300
400
600

Figure

Click here to download Figure S2_FigA.tif

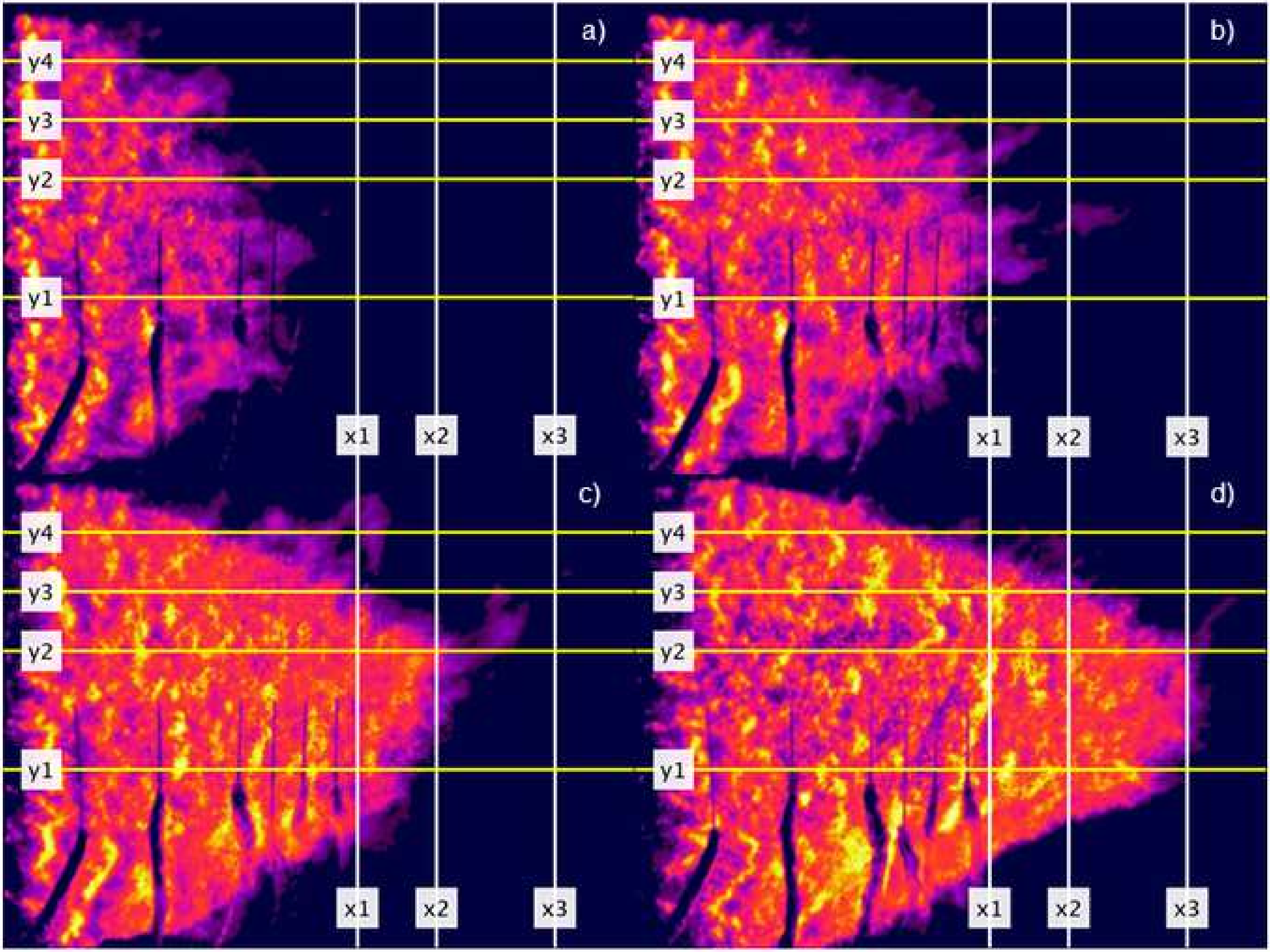

Click here to download Figure S2_FigB.tif

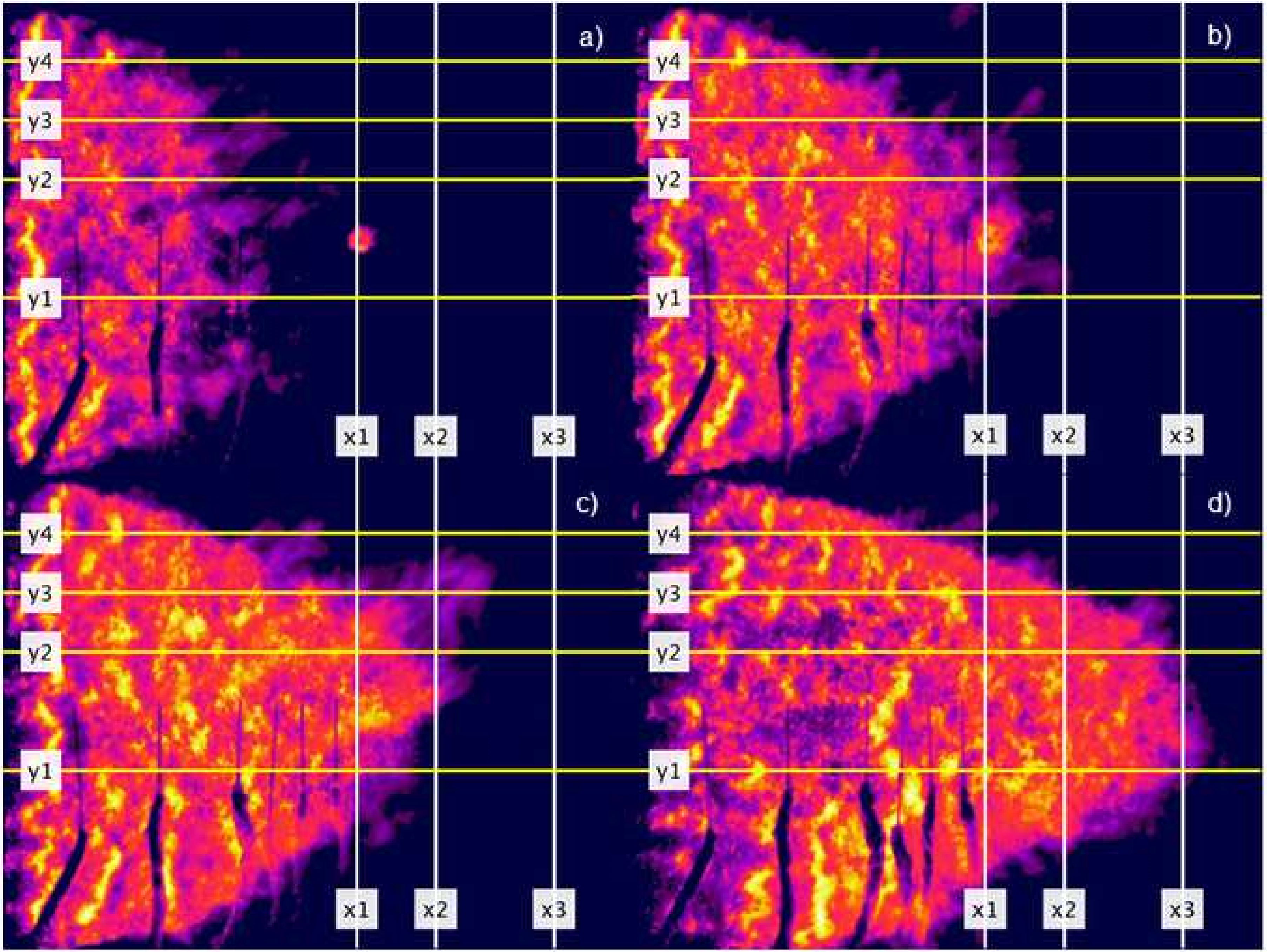

Click here to download Figure S2_FigC.tif

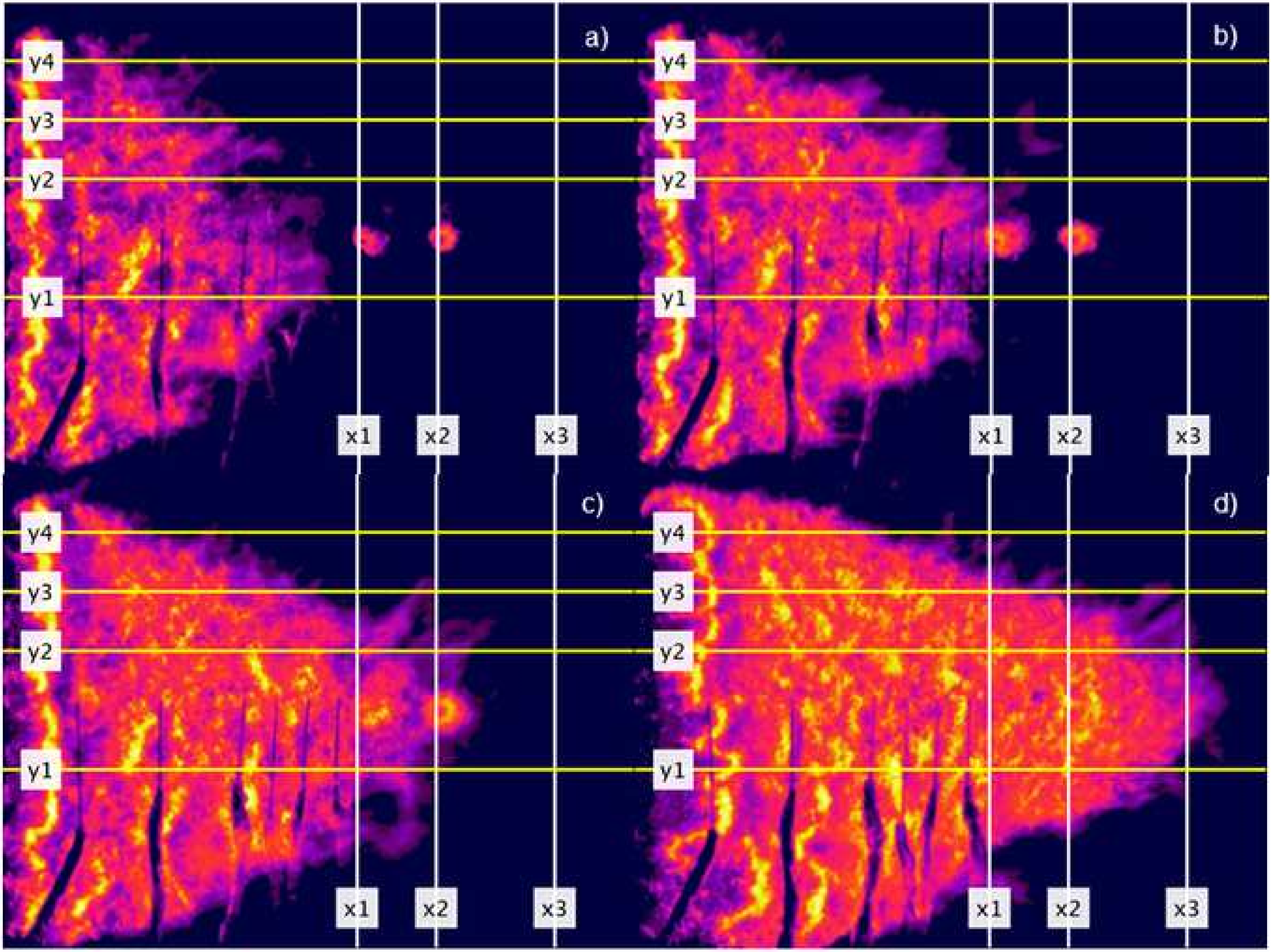

Figure

Click here to download Figure S2_FigD.tif

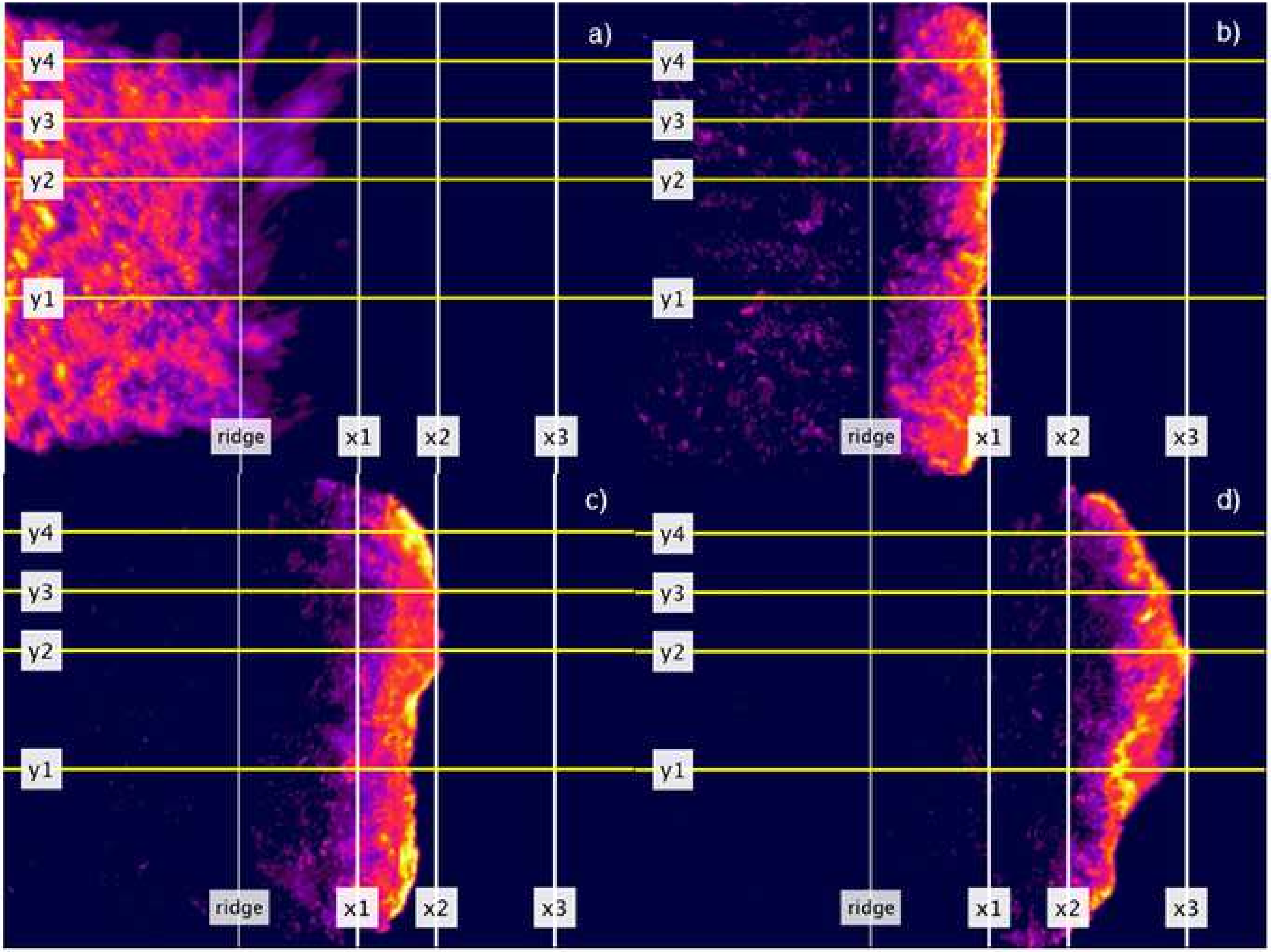

Figure

Click here to download Figure S2_FigE.tif

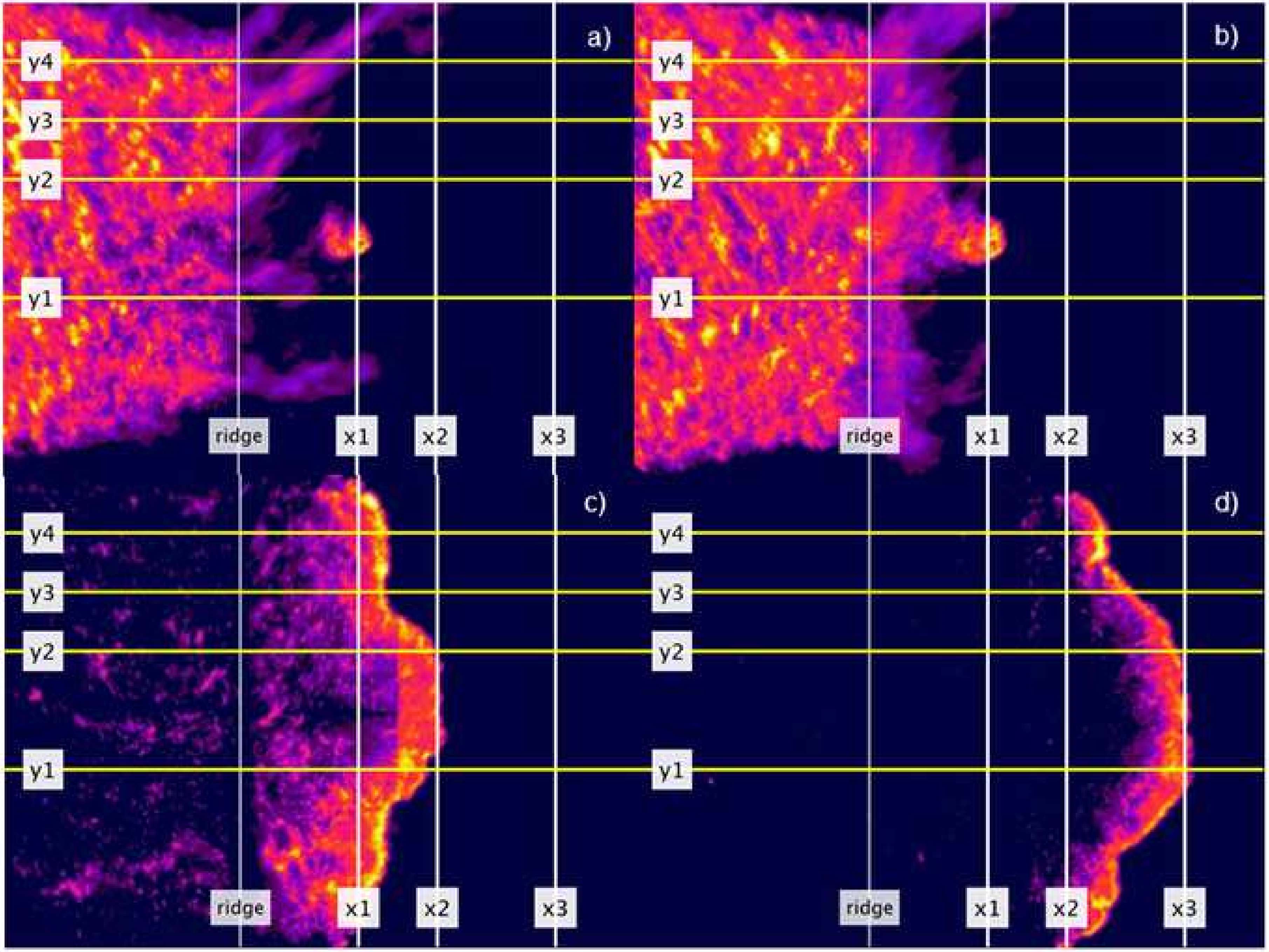

Figure

Click here to download Figure S2_FigF.tif

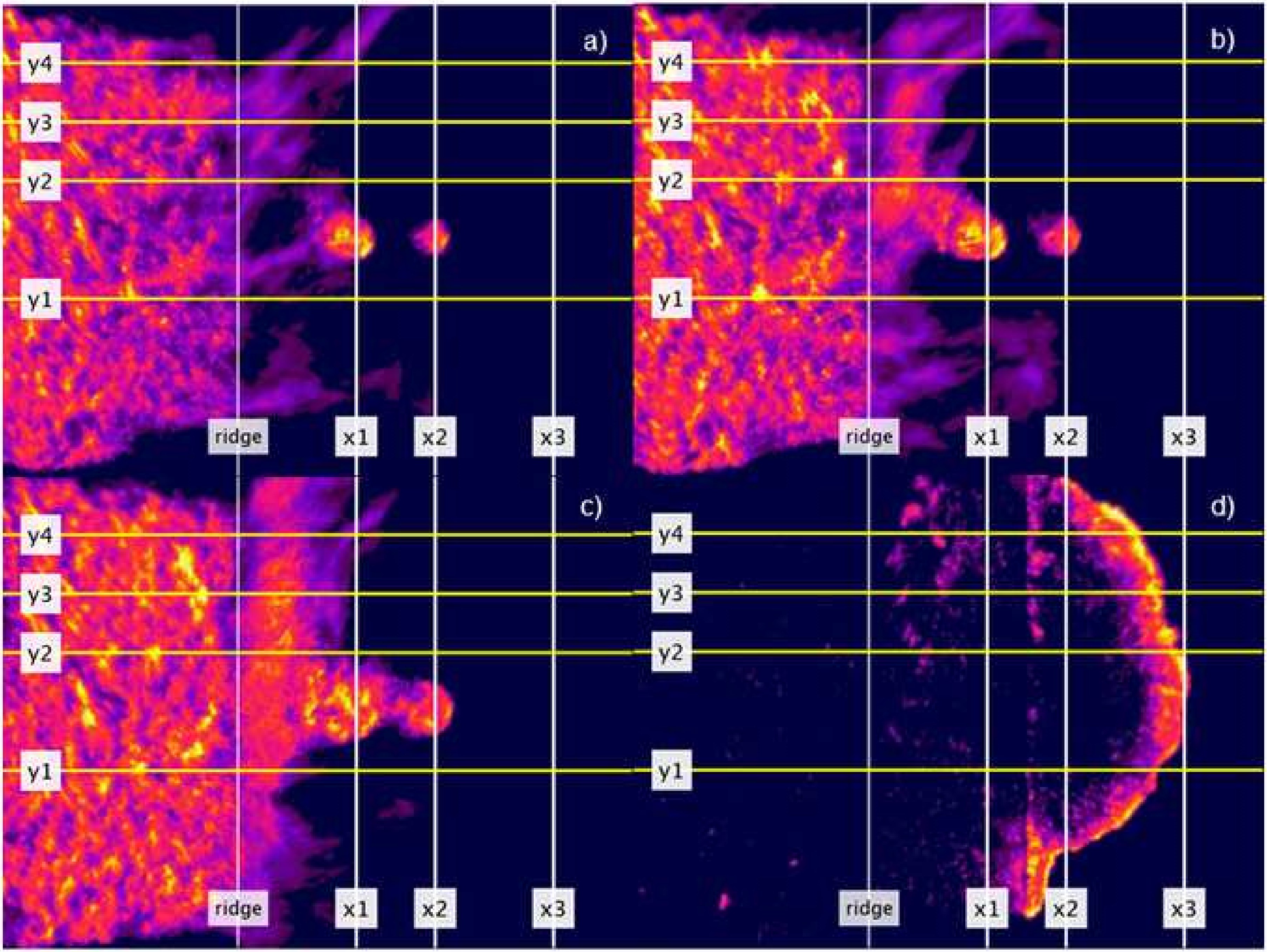

Click here to access/download
Supporting Information
S1_Appendix.docx

Click here to access/download
Supporting Information
S2_Appendix.docx

Click here to access/download
Supporting Information
S3_Video.mp4

Click here to access/download
Supporting Information
S4_Video.mp4

Click here to access/download
Supporting Information
S5_experiment_data_head_fire.csv

Click here to access/download
Supporting Information
S6_experiment_data_offcentre_fire.csv
