# Supplementary material for: Experiments on the influence of spot fire and topography interaction on fire rate of spread
Source: PLoS One. 2021 Jan 7;16(1):e0245132. doi: 10.1371/journal.pone.0245132 (PMC7790231; doi:10.1371/journal.pone.0245132)
Supplement: Attachment — Submitted filename: Responses_to_reviewer_comments.docx [file pone.0245132.s008.docx]

We thank all the reviewers for the time they have taken to review our work and provide comments. We have organised our responses to the comments below. The reviewer comments are included here in the red italic text, with our responses in the black text, grouped underneath each reviewers’ comments.

Reviewer #1: General comments
--This paper details experiments investigating the role of spot fires in influencing the behavior of fires burning over broken topography. Small-scale experiments in a laboratory setting are utilized to investigate the effect of a small number of variables. While the topic of this ms is of broad importance in the world of wildland fire science, unfortunately the approach taken by the authors and their treatment of the problem is little more than a cursory exploration of the phase space of possibilities with little regard for implication in the broader context of wildland fire behavior.

--Critical here is the experimental design and the concept of ‘combined’ fire rate of spread, both of which indicate a lack of thorough consideration of the problem in developing the research question into a meaningful experiment. It’s not clear if the observation in the field upon which this work is based of wildland fire rates of spread increasing with spotting are the result of merging of spot fires with the main fire, or independent propagation of the spot fires, forming a pseudo-front that then is considered the head of the fire. More often than not, spot fires are overrun by the main fire unless the main fire is held-up for some reason (break in topography or fuel, etc). The approach taken here seems to assume the former option, but if this is the case, then the experimental design seems intended to not understand the key variables driving this interpretation. If the speed of a fire is defined only by its progression of a flaming edge regardless of its speed, then slowly moving but separated point fires may be seen to ‘advance’ the fire once they join but only by the distance of the location of the spots since they don’t spread very far and not their rate of spread. As a result, the location of the spot fire and its speed of connection with the main fire then become the critical variables, neither of which are investigated in this study. Interactions of the spot fires with main fire also do not seem to be of much interest given the method of calculating cumulative ROS at three locations. As it stands, the current experimental design is largely a matter of geometry rather than fire behavior and suggests that there is an optimal distance and time for spot fires to merge and increase your ‘combined’ ROS. In my mind there is a fundamental difference between spot fires that actively spread, advancing the fire area, and spot fires that merge but don’t spread but inadvertently increase ‘combined’ ROS.

Furthermore, the experimental design does not consider a meaningful control against which experiments may be compared. Use of a zero wind, zero slope rate of spread of a fire burning on a 1 m^2 area is not meaningful as in both cases (wind and/or slope) the flame propagation mechanism is different to the zero wind and zero slope case and we know that larger fires spread faster. In this work, the control could be the no-spotting case, with and without a hill.

The use of a single hill with arbitrary slope and height does not provide any insight into the effect of a hill on rate of spread as it is not a variable and raises far too many questions for a publication of this type. Why were the values chosen? Were others considered? Why is the hill asymmetric? Why this asymmetry? Similarly, why 1.5 m/s wind? This is also a very important variable that is not recognized or studied appropriately, as is fuel moisture content. Too much of the methodology appears arbitrary without due consideration of the types and ranges of variables involved. Much of the analysis of results is superficial and inference of results in the discussion and conclusions do not adequately consider the limitations of the study. Furthermore, much of the discussion is filled with speculation without any attempt to isolate or specify probable influence.

The documentation of methods utilized in this study is woefully inadequate with insufficient detail to enable others to repeat your experiments. What was the length of the ignition line? How was the flame front defined in the infra-red? What were the ‘similar weather conditions’ of the experiments? Experiments conducted in sets of threes is not random.

The structure of the paper mixes methods and results throughout. A thorough rethink on the contents would improve the logic and flow of the paper. It is not until deep into the discussion that it becomes clear that the primary mechanism of spot fires influencing fire spread is that they spread backward against the main wind direction, which alters the entire perspective of the experiment, its results and implications. The reliance upon speculation of the presence of a lee-slope rotor further weakens the results.

Other general questions that arose: Why were spots allowed to grow to a predetermined size and what relation was this to the time of ignition of the main fire? Why was the wind turned on only after the main fire was ignited? In reality the spots would be ignited after the main fire reached the ridge. What effect did this method have on results? What was the measurement error of the results? Did the FLIR analysis method change with FMC? In the results, what is the effect of fire spread and what is the effect of geometry? Would the results of one spot fire at spot fire location 2 have produced any different result?

Thanks to the reviewer for their comments. In the paragraphs below, we have grouped the responses to the various issues raised:

- An issue that the reviewer raises is it that alternative levels of variables were not explored in the experiments and that key drivers, which they state as “the location of the spot fire and its speed of connection with the main fire”, have not been investigated. We agree that these are important areas to explore and there would certainly be room to extend these experiments in the future, which we mention in the discussion: e.g. test other slope angles, spot fire numbers and arrangements, wind speeds, different spot fire ignition patterns, analyse speed of connection etc. However, exploring these are beyond the scope of our study. We conducted this study to investigate broad differences how spot fire(s) affect the overall spread of a fire (i.e. our “combined” rate of spread) over different terrains. We are not aware of any published studies that focus on this topic, thus it was important to design and conduct experiments to test distinct conditions (i.e. steep hill and no hill) to establish whether broad differences in spread exist or not, and understand whether this is an area in which further investigation is warranted. While the experimental set up and conditions were designed to be simple, they clearly demonstrate an important effect that (as far as we are aware) has not previously been documented in published studies.
- Also in relation to our limiting of variable ranges (e.g. one wind speed), a strength of our approach is that each experiment set up was replicated 5 times, which allowed for robust statistical analysis. In such experiments, it can always be expected that there will be small non-systematic differences in the conditions which may have affected rate of spread, but which are too difficult to quantify. This may include small variations in depth across the fuel bed, variation in the fire line ignition by a few centimetres, or small differences in visual interpretation of image. However, the repetition of each experiments means that, despite these small variations, the major effects of spot fire number and hill/no-hill will come through in the statistical analysis. Due to the need for repetitions, and inevitable limitations on time and budget, it was necessary to limit the number of unique experimental conditions. Thus, we chose to investigate large difference in spread between two distinct topographies of flat fuel bed and steep hill, and zero to two spot fires.
- The reviewer argues that wind speed and fuel moisture content were not recognized or studied appropriately. Both of these were considered in the design of the experiments: wind speed was set to the same level for all experiments and the effect of fuel moisture content was controlled for through our use of the relative rate of spread. It would be interesting in future experiments to test and isolate effects of different wind speeds and FMC, but this was beyond the scope of our study, particularly given our need for 5 replicates of each experiment set up. Adding in one more alternative wind speed would require double the amount of experiments. We had time and budget to run 30 experiments, so needed to limit the number of variable combinations. Running say experiments with 4 wind levels, 4 spot levels and 4 slope levels, each with 5 repetitions, would require 320 experiments.
- The reviewer’s overall opinion of the methods sections is that it is inadequate. However, save for some small oversights that we have corrected, we argue that our description of the experiments and analysis is sufficient, given (a) two out of three reviewers were happy with the methods section (reviewer 2 said “I commend the authors on a well-planned and executed experiment”) and (b) that there is a similar level of detail in our manuscript to comparable laboratory-based studies of fire spread. For example, the reviewer asks: “How was the flame front defined in the infra-red?”. We describe our method in the paragraph beginning line 292: “Times to cross each line were identified through visual interpretation of the rectified FLIR images...”. This description is similar to comparable studies, for example, Sullivan et al. (2019) and Butler et al. (2020) describe simple “ocular” and “visual observation” based methods on identifying the flame front from video recordings to measure rate of spread. Moreover, we provide more detail on our visual method, as we have provided several examples of actual images and fire crossings of measurement interval lines for the reader: i.e. Fig 5 and 6 in the main body, and the six figures in Appendix S2.

While we have not made any large changes to the methods section in the manuscript, we have added some clarification text in the manuscript in response to some specific issues raised:

- Fire ignition line was the entire width of the fuel bed (300 cm).
- Spot fires were allowed to grow to a predetermined size to ensure they were established in the fuel bed.
- Fan was turned on after the fire line was ignited, so the entire fire line was established in the needles below the thread. This was also the method in similar studies (Butler et al., 2020; Raposo, 2015)
- Values for fuel load, wind and slope were chosen as moderate values within the range used in previous experiments in the lab. We have added clarification text to the manuscript.
- An asymmetric hill was used due to is availablity from previous fire behaviour experiments in the laboratory (Raposo, 2015). We have added this to the text.
- The reviewer argues that the effects in the manuscript are “largely a matter of geometry” and not of fire behaviour. It is unclear if the reviewer is arguing that the effects on ROS we describe are not important, or whether our description needs to be altered. In any case, the effect we investigate and describe is important, as it helps to understand the way wildfires move across a landscape. We do consider the differences in ROS we identify to be a matter of fire behaviour, as in operational wildfire situations, the overall movement of fire across a landscape (main fire and merging spots) is the important measurement, with details of how spot fires connect with the main fire not the focus. Responders mainly need to know when the main burning area will reach a community/cross certain areas. However, given that reviewer 2 has also mentioned a lack of clarity in our description of ROS measurements for both the spot and main fires, we have updated our description in the manuscript for clarification (see paragraph starting L63, and starting L226).
- The reviewer says that the manuscript mixes methods and results throughout. This appears to be referring to the mention in the methods section of the fuel moisture content, relative humidity and temperature during the experiments. We argue that this is reasonable to include in the methods section, as it describes the conditions under which the experiments were conducted and are not the results of the experiments (i.e. the rates of spread).
- The reviewer mentions that they thought it was unclear that there was upslope spread of the spot fire. In the General Observations section of the results, we have now expanded the sentence where we mention the upslope spread of the spot fires during the hill-present experiment to make this clearer. We have also added an additional Figure (now Fig 7), which is a series of still images from a side-on RGB camera that shows the up slope spread of a spot fire.

Specific comments
Lay readers you need to define many terms such as spotting at first use. In particular, ‘combined ROS’ needs a robust definition.

We have added to the descriptions of spotting and combined ROS in the first two paragraphs to provide clearer definitions.

L85-86: This statement is a truism.

Our statement here: “*Depending on local combinations of these (and other) factors, it is likely that similar levels of spotting could significantly influence the combined ROS in some wildfires but not others*” would not be completely obvious to every reader. Some may think that similar levels of spotting would produce similar results in ROS. This sentence describes the focus of this paper, so we think it is reasonable to include.

Table 1: Slope angles should be relative to wind direction (windward = positive, leeward = negative).

We have removed the slope angles from the table, as these aren’t necessary in the table and are better described in the text and by referring to the schematic figures.

L151: Why was 0.8 kg/m^2 selected? It seems rather light in regard to wildland fuel loads. How is it representative?

0.8 kg/m^2 allows for continuous fire spread across the fuel bed, rather than patchy/clumped spread that can occur in lighter fuels. This load is within the range that such experiments are commonly conducted in other studies including in the same laboratory: slightly more that Raposo (2015), less than Sullivan et al. (2019) and about the same as Butler et al. (2020). We have added some extra text in the manuscript to clarify:

*“This dry weight of fuel is above the threshold that allows for continuous fire spread, rather than patchy fire spread that has been found in previous experiments in the same laboratory at lower fuel weights (Raposo, 2015).”*

L155-160: This section is not clear and confusing. Results should be kept for results section.

This section has been updated to make it a bit clearer. In regard to the final sentence of this section, this is simply a description of the conditions (FMC, humidity etc.) under which the experiments were conducted, which we think is appropriate for the methods section.

L162: SI units.

This comment appears to be referring to the model hill width, where we say “the model hill was 400 cm wide...”. We believe using “cm” here is the correct units.

L167: Did the rounded ends work? What effect did they have on the overall experimental set up?

The sentence has been updated to provide some more explanation here: “*The model hill itself has no coverings installed at each end (Fig 2a), thus would be open to air flow and the creation of excessive turbulence at the hill ends. To better represent an actual hill, rounded ends were added to smooth the air flow around each end of the hill (Fig 2b). For the hill absent experiments, the fuel bed was simply a 300 cm width x 400 cm length flat surface (Fig 4).”*

L191: Why not use a control?

Our method used here means that variation in FMC between experiments is accounted for, and allows for statistical comparison between all the unique experiment set ups using the two-way ANOVA. This would not necessarily be the case if comparing treatment experiment results to control experiment results, as the FMC of the control experiments would be different to the FMC in treatment experiments. Also, using the two-way Anova and Tukey’s tests, the ROS of the one and two spot fire experiments are compared to the zero spot fires experiments anyway, without the need to designate these as the control, as suggested earlier.

L211-213: These are not interval rates of spread but cumulative rates of spread. An interval rate of spread would be L1-L2 and L2-L3. Actual interval rate of spread could make for interesting analysis.
Figure 5 and 6: Put times into the figures.

Describing this as cumulative ROS would not be appropriate here, as cumulative implies summing ROS measured at separate intervals: e.g. (L0 to L1) + (L1 to L2) + (L2 to L3). Describing what we have measured in the manuscript as intervals is correct, as we have measured ROS between two distinct points. They are still intervals but just overlap.

L239: Show this to be the case.

We argue that our assertion in the manuscript is reasonable, given the small amount of variability (a few centimetres within a 400 cm length fuel bed) and that each experiment set up was replicated 5 times each, meaning the main effects come through in the statistical analysis despite small variations in experiment conditions. We have added a clarification here: “...*given that experiments with each unique combination of factors were replicated 5 times...”*

L244: What sort of confounding effects? Should they be present at all for these experiments if they affected the flow? Did they affect ROS? Show this.

We have added some more explanation here. This section now reads:

*“Off-centre ROS was measured only on the side of the fuel bed without pitot tubes and pipes (installed for a separate analysis). This was because there may have been some minor slowing of fire spread on the flank with the pipes, which ran 15 cm above the surface of the fuel bed. This should be noted when interpreting the results, although our observations during the experiments suggested any effect on the spread of the head fire and flank fire on the opposite side to the pipes was negligible.”*

Table 2: These results show that the cumulative rate of spread for all experiments (with the exception of Hill-absent 2-spots decreased across each measurement. Why was this the case? Indicates the fires continued to decelerate and thus were not in equilibrium, even without slope and spot fires. Present standard deviations.

We have added standard deviations to Table 2 now, and have mentioned the observation of some deceleration now in the results:

*“For each unique set of conditions, head fire R’ was generally fastest when measured over the Line 0 – Line 1 interval, and slowest over the Line 0 – Line 3 interval, indicating some deceleration in fire spread across the length of the fuel bed.”*

We are not sure of the exact reason for this, perhaps after an initial surge in spread close to the fan, there was deceleration further away from the fan. Alternatively, the centre of the fire line was the hottest, and thus spread fastest, which formed the ellipse shape, at which point the spread rate slowed somewhat. However, we view this as a minor point in comparison to our focus in the study, which is the broad differences in ROS between spotting and hill absent/present cases. Given that our comparison was between ROS at the same intervals across the experiments (e.g. L0 to L2 ROS in hill-absent 1 spot experiments was compared to L0 to L2 in in hill-present 1 spot experiments), this observation did not affect our main findings.

L384: No, it doesn’t. You considered one topography and two fixed points which showed these to not be a major factor in affecting fire behaviour.

Our sentence here is justified as it is based on our results. We state “*Our results provide strong evidence that topography can be a major factor controlling how spot fires contribute to fire spread*”, which is supported by the results. We identified statistically significant differences in combined ROS between the flat fuel bed and fuel bed with a hill, thus topography can be a major controlling factor.

L392: Did you measure differences in air flow? Did you quantify fire-fire interactions? Where is the data for spot fire growth prior to merging?

These are beyond the scope of the current study. The main interest was the broad differences in fire spread considering differences in topography and spot number. We argue it is reasonable to mention fire-fire interaction, air flow differences etc. in the discussion as possible influences, to give the reader some wider context. A more detailed analysis could be conducted of other experiments in the future, although the next step may be to look for the broad patterns described here in real wildfire data.

L394: How large did a spot fire need to grow to influence head fire behavior?

This was not analysed in our study, as we focused on the differences in overall/combined fire spread.

L395: Pure speculation.

The sentence has been deleted as it is not needed.

L401-402: This observation needs to be quantified and presented in results. At what speed did this occur?

Our focus was on the broad differences in the overall fire spread under different topography and spot fire numbers. We mention in the first paragraph of the results that our observation was of predominantly upslope spread of the spot fires, and we have added an extra figure (Fig 7) here too for clarity. This is a general observation that helps explain the results, but we don’t believe a detailed analysis is necessary to meet our aim in this paper. Detailed analysis of the rate of merging could be conducted in future studies, ideally, if the results are to be applied in wildfire models, using actual wildfire observations (e.g. line scans). As two of the three reviewers have not raised this issue, we have not made changes here.

L408: How common are these? What happens when they are not present? Show that they were important here and not mere speculation.

In response to this and a below comment, we have updated this section to a more general discussion about possible influences here:

“While fires are generally expected to travel faster upslope, other factors are likely to have contributed to this observation. The behaviour may have partially been the result of fire-fire interaction, where the smaller spot fire(s) were drawn towards the larger line fire due to indraft effects (Finney and McAllister, 2011). The behaviour may also be explained by the possible formation of a lee-slope rotor, rather than a laminar air flow: i.e. as air flow separated from the surface once past the ridge, a circulation of air that flowed from the base to the top of the lee slope may formed, similar to what has been described in previous experiments in the same laboratory with similar ridge and wind conditions (Raposo, 2015). Although we have not analysed the drivers of the upslope spread here, the result is that the combined fire was able to overcome the low spread potential of the downslope: i.e. the spot and main fires merged into a larger combined fire, with the new fire front extending to the farthest downwind point of the original spot fire.”

L411-412: This is not what happened as it was just a matter of geometry.

This section has been changed in response to the previous comment. Overall though, our comment is accurate. The merging of the spot fire and the main fire meant that the combined fire overcame the area of low spread potential (the downslope), and the resulting combined ROS (depending on number of spot fires) could be similar to no hill conditions. This may be important insight into real spotting during wildfires, where the overall spread of the fire is the important measurement, not individual rates of a main fire and each spot fire.

L417: Speculation.

This is one possible explanation for our results, thus we have added “A possible explanation” to the start of the sentence: “*A possible explanation for this is that fire-fire interaction and merging of spot fires may have generated greater radiant heat, which led to more rapid drying and ignition of nearby fuels (i.e. in the off-centre measurement zone), thus faster R’.”*

L423: Define what you mean by ‘more curved shape’.

We have clarified this to now read: “*In all the 2 spot fire hill present experiments, the fire front had a much more curved shape due the extension (and combination of downwind and outward spread) caused by the merging of the 2 spot fires (e.g. Fig 6), compared to the no spot fire hill present experiments where the fire front was relatively flat (e.g. S2 Appendix Fig D)*”

L426: Speculation.

Junction zone behaviour has been found to be important in some other studies, so it is worth mentioning that this may have had some influence here. This may be an important factor in future studies considering real wildfire data, or other experiment studies.

L428: Small scale but also narrow set of conditions and variables.

Our sentence here is *“It is important to consider how the results from our experiments, under a narrow set of conditions, may apply to actual wildfires.”* , which already mentions the issue. No change has been made in relation to this comment.

L446-454: Relevance of this paragraph?

The main purpose of these experiments is as a starting point to look at how spot fires and topography interaction affects fire spread, with the eventual goal of understanding how this works in real wildfires. The purpose of this paragraph is to give readers context of how the effects we describe may be important in real fires, as this is the eventual goal. We don’t know the precise implication to wildfires from the experiments, but it is useful context. Future studies are needed to look to see if the behaviour found in the experiments does actually occur in real wildfires.

L463: Relevance?

These are discussion points around how spotting and topography may interact in real wildfires, in particular one way that spotting may be important to fire spread. It is important to give this context for our results, and to point out that spread in wildfires may be more complex. This specific section is saying the effects may be different if there are more spot fires. We think it is important to discuss these points, as future studies will need to investigate spotting/topography and fire spread using real wildfire data, such as line scans.

L491-493: This is not a new finding.

We have used the word “confirmed” here instead of ‘demonstrate’ to convey this is not necessarily a new finding.

L497: By how much?

This is a general point based on experiment results. We could not specify by how much for real wildfires, this would need investigation using real wildfire data.

Reviewer #2:

This is a review of the manuscript entitled “Experiments on the influence of spot fire and topography interaction on fire rate of spread” submitted to the Journal PLOS ONE. The authors describe a controlled and replicated experiment to better understand interactions between terrain and spot fires on fire rate of spread. I commend the authors on a well-planned and executed experiment. The mechanisms by which spot fires contribute to wildfire spread, and how that is incorporated in larger landscape-level fire simulation models is not well understood and this research helps make incremental steps towards that goal. The limitations of the experiment were well described in the manuscript and future steps forward were also addressed. The main comment I have is that the study captures only one dynamic of fire activity (i.e., ROS), but misses other important aspects of spotting that can’t really be observed at such a small scale. In terms of spotting, the most important thing really isn’t ROS, per se, it is the ability of fires to jump fire lines or other natural features that would otherwise be successful barriers to spread. It is the ability of fires to act as ignition sources and create several flaming fronts that can erupt meters to kilometers from the main fire front. The authors should put their results in the context of broader implications of spotting on fire suppression and fire modeling efforts.

Comments:
I very much like the use of the relative rate of spread as the response variable, which accounts for temporal variation in moisture conditions as opposed to raw rate of spread.
It seems as if there was quite a range of environmental conditions: (Lines 158-160) “Over the 30 experiments fuel moisture content ranged between 11% and 16%, relative humidity and temperature (measured inside the laboratory) were between 30% and 79% and 19oC and 29oC, respectively”. It would be good to see the distributions of these variables across the different replicates. Were the hill experiments hotter and drier consistently than the flat slope runs? I get that the R’ is probably the most important aspect of the combustion spread, but it’s really the variability within treatments that is interesting and hasn’t been explored and looking into the environmental variables could help.

It is likely my misunderstanding of your methods, but it’s not clear how ROS is actually accounted when taking into account the spot fire. For instance, what if the spot fires crosses the end of the fuel bed before the fire started at line 0 reached line 1. How would R’ be calculated then?

For such a controlled experiment there appeared to be high variability in R’ within a treatment (i.e., across replicates, Fig 7, 8). Can this variability be explained by variations in local weather during the runs?
What was the variability in R0? Were there big differences across experimental treatments? Perhaps this could be included in the supplementary material. In general, it would be good to know how much the variability in external weather conditions likely influenced model results.

Thanks to the reviewer for their comments. Our responses are grouped in the dot points below.

- In response to the above points about spotting jumping barriers, we’d say that one of the arguments in that paper is that in addition to spotting jumping barries, that spotting may be very important for the overall ROS of a wildfire in some cases (e.g. hilly areas), and that this is something that needs attention by researchers. So, we think it is worth mentioning how spotting affects suppression and jumps barriers etc. but it is not the main focus here. We have mentioned how these results might be important for ROS modelling efforts in the discussion already, but we have now added an additional sentence in the introduction regarding suppression: *“Spotting can easily jump fuel breaks such as roads, which can substantially reduce the chance of a wildfire being contained by fire crews.”*
- In response to the point about how we measured ROS (i.e. R) and R’, we measured ROS as it would be measured in an operational wildfire situation, where spot fires are treated separately, but once they have merged with the main fire, all the merged area is mapped as the wildfire. There is not always a clear distinction, as there may be cases with lots of separate spotting where you may want to treat the whole area as the fire front. We have updated the description of combined ROS in the introduction and methods, and also where we discuss combined ROS in the discussion. We hope this clarifies what we are talking about here. In the particular case of the spot fire reaching the end of the fuel bed first, according to our definition, the spot fire would not impact ROS because it had not merged with the main first. We would not expect this to happen in real fires (i.e. a spot spreading so much faster than the main fire), unless there was a no fuel area between the spot and main fire. If this did happen in the experiments, we think we would need different experiments, perhaps with a larger fuel bed, to understand the phenomenon.
- In response to the points about environmental conditions, there was no clear difference in FMC between the hill present and flat fuel bed cohort, so this is unlikely to have affected our results. We have added the FMC measured prior to each experiment as a column in the supplementary data file S5 and S6, and added this text in the methods: *“Fuel moisture content was between 11.1% and 16% over all experiments, and ranged between 11.1 % and 16 % for the flat fuel bed experiments (mean 13.7 %), and 11.5 % and 15.3 % for the hill-present experiments (mean 14 %).*”
- We suspect small differences in fuel depths across the fuel bed between experiments, small differences in the manual ignition method, switching the fan on etc. may have contributed to this variation between individual experiments. However, we believe our analysis is robust due to having 5 replicates of each experiment set up, which allowed for our main effects of interest to come through in the statistics. Detailed analysis of the variation between experiments was beyond the scope of the work here, as we focused on large differences in ROS between hill/spot number conditions. This is something that could be looked at in future experiment work, although we see the next step after this work as looking for evidence of the influence of spotting on ROS using actual wildfire data.

Line 423: should read “do to the extension”

This sentence has been updated

Reviewer #3:

It is a very interesting paper addressing an important issue. The title reflect the content of the manuscript. The methodology and analysis are sound . The weaknesses of the experiment are identified by the authors. The manuscript is well written with clear language.

Thanks to the reviewer for their comments.

In response to the comments in the pdf:

1. We have removed the reference to “other factors”, as it is not necessary to make the point.
2. We have added clarification in the methods around our selection of a 1.5 m/s wind speed.
3. We removed the phrase “- ROS on flat surface without wind”. This was referring to the conditions in the basic ROS tests on the 1 x 1 m fuel bed. But we think now it is not necassary to explain this again in the results, so now there is just a simple mention of “*R_0_*”, which is explained in the methods.

**References used in our responses**

Butler, B., Quarles, S., Standohar-Alfano, C., Morrison, M., Jimenez, D., Sopko, P., Wold, C., Bradshaw, L., Atwood, L., Landon, J., O'Brien, J., Hornsby, B., Wagenbrenner, N., Page, W., 2020. Exploring fire response to high wind speeds: fire rate of spread, energy release and flame residence time from fires burned in pine needle beds under winds up to 27 m s−1. International Journal of Wildland Fire 29(1) 81-92.

Finney, M.A., McAllister, S.S., 2011. A review of fire interactions and mass fires. Journal of Combustion 2011.

Raposo, J.R., 2015. Experimental analysis of fire spread across a two-dimensional ridge under wind conditions. International Journal of Wildland Fire 24(7) 1008-1022.

Sullivan, A.L., Swedosh, W., Hurley, R.J., Sharples, J.J., Hilton, J.E., 2019. Investigation of the effects of interactions of intersecting oblique fire lines with and without wind in a combustion wind tunnel. International Journal of Wildland Fire 28(9) 704-719.
